# The role of vanadium substitution in the oxygen sublattice disorder of Ba_7_Nb_4_MoO_20_-based hexagonal perovskite oxide-ion conductors†

**DOI:** 10.1039/d4ta01540a

**Published:** 2024-09-25

**Authors:** Abdulkadir Olatunbosun Biffo, Theodosios Famprikis, Pedro B. Groszewicz

**Affiliations:** a Department of Radiation Science and Technology, Delft University of Technology Delft 2629JB Netherlands t.famprikis@tudelft.nl p.groszewicz@tudelft.nl; b Helmholtz Zentrum Berlin für Materialien und Energie Berlin 14109 Germany

## Abstract

Ba_7_Nb_4_MoO_20_-based hexagonal perovskite derivatives are promising oxygen-ion conductors for solid electrolytes in solid-oxide fuel cells and electrolysers. A thorough understanding of chemical substitution and its impact on structural features conducive to high ionic conductivity is fundamental for decreasing the operation temperature of such devices. Here, a new 7H polytype-based composition, namely Ba_7_Nb_3.9−*x*_V_*x*_Mo_1.1_O_20.05_, is investigated to assess the effect of vanadium substitution. Structural changes upon V incorporation are studied using X-ray and neutron diffraction, as well as ^51^V and ^93^Nb solid-state nuclear magnetic resonance spectroscopy. For the undoped composition at room temperature, two distinct oxygen sites (O1 and O5) are found along the palmierite-like layer, corresponding to a mix of four- and six-fold coordination for adjacent M2 cations. At high temperature (527 °C), reorganization of oxygen results in the major occupation of O1 and four-fold (tetrahedral) coordination of the M2 cations. The same rearrangement is observed upon V-substitution, but already at room temperature. From ^51^V NMR, we identified a tetrahedral coordination for V^5+^ cations, indicating their preferential occupation of the M2 site. This preferential occupation by V^5+^ cations is correlated with increasing tetrahedral coordination of Nb^5+^ cations as observed from ^93^Nb NMR. Altogether, these observations indicate that V-substitution impacts the oxygen sublattice so as to mimic the high-temperature structure. Additionally, BVSE calculations demonstrate a decreasing energy barrier for O^2−^ migration associated with the presence of vanadium in the structure. This conclusion corroborates the hypothesis that vanadium's propensity for a lower coordination number is beneficial for promoting high O^2−^ mobility in this promising class of oxide-ion conductors.

## Abstract 2

We track the evolution of the oxygen sublattice disorder in hexagonal perovskite oxides upon vanadium aliovalent substitution using neutron, X-ray diffraction and solid-state NMR spectroscopy.

## Graphical abstract

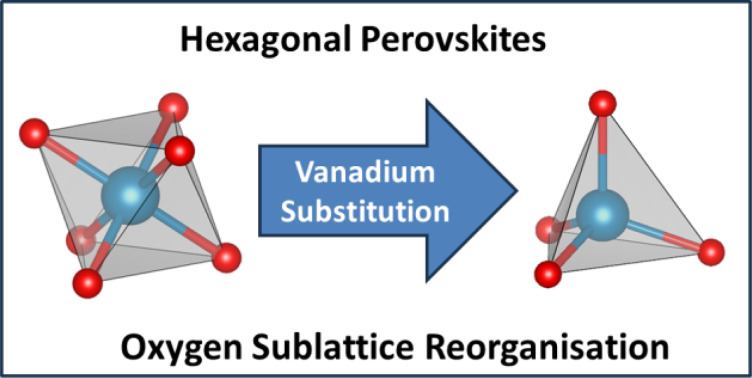

## Introduction

Solid oxide fuel cells (SOFCs) are promising energy conversion devices to propel the transition towards a fossil-fuel-free energy economy.^1,2^ The widespread use of SOFCs is hampered by their high operation temperatures, in the range of 800 – 1000 °C for current commercial devices based on yttrium-stabilised zirconia as solid-state electrolyte.^3^ These high operating temperatures pose considerable challenges, such as the degradation of metallic interconnects and slow start/stop times, as well as imposing restrictions on the selection of compatible materials.^4,5^ Given this context, it is imperative to lower the operation temperature of SOFCs to the so-called intermediate temperature range of less than 600 °C.^6^ Doing this will require finding a suitable electrolyte, which is the first step towards enabling the efficient operation of SOFCs in the intermediate temperature range. The fine-tuning of the most widely used electrolyte materials, such as fluorite-type oxides, and the use of increasingly thinner membranes are saturated strategies.^7–11^ Hence, there is a need to explore alternative structures or materials. Several families of oxides have been proposed as alternative structures for oxide-ion conduction such as silicon and germanium apatites (La_9.33_Si_6−*x*_Ge_*x*_O_26_),^12^ Bi_4_V_2_O_11_ compounds,^13^ La_2_Mo_2_O_9_ (ref. 14 and 15) and LaSrGa_3_O_7_ materials.^16^ Recently, a high oxide-ion conduction at the intermediate temperature has been reported in hexagonal perovskite derivatives.^17–22^

**Fig. 1:**
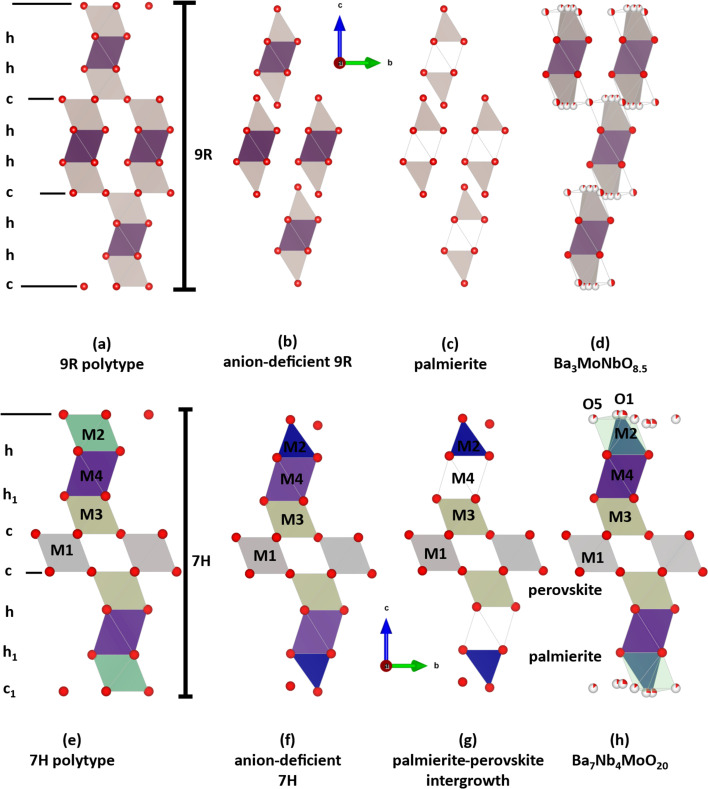
The *cb* plane projections depicting the relationship between (a) 9R hexagonal perovskite polytype and palmierite-type derivative structures; (b) anion deficiency and partially-occupied face-sharing octahedra; (c) palmierite structure with empty octahedra; (d) the palmierite-perovskite intergrowth in Ba_3_NbMoO_8.5_ reported by Fop *et al.*^31^ This image is adapted from Mitchell.^27^ The *cb* plane projections illustrate the relationship between (e) 7H hexagonal perovskite polytype, (f) oxygen-deficient c_1_ – BaO_3_, and (g) unoccupied h_1_ octahedral units.^32^ And (h) the structure reported by Fop *et al.*^22^ Colored polyhedra denote the different crystallographically equivalent metal-cation sites.

An example of a hexagonal perovskite family that has shown a high oxide-ion conduction, comparable to yttrium-stabilised zirconia, is the Ba_3_M’M”O_8.5_ series (where M’ = Mo, W and M’’ = V, Nb).^21,23–26^ This series is a palmierite derivative of the 9R polytype of hexagonal perovskite depicted in Fig. 1(a–d).^27^ The bulk conductivity of a member of this series Ba_3_MoNbO_8.5_ has been reported to be around 2.2 mS cm^−1^ at 600 °C, predominantly dominated by oxide-ion conduction,^26^ which has been evidenced using the bond-valence site energy (BVSE) approach to proceed *via* a 2D migration path involving the partially occupied oxygen sites in the palmierite-like layers.^21,28–30^ Furthermore, the conductivity of this palmierite derivative has been improved by almost an order of magnitude *via* an iso-valent substitution of Nb^5+^ with V^5+^,^21^ with a bulk ionic conductivity of 11 mS cm^−1^ at 600 °C reported for the composition Ba_3_Nb_0.9_V_0.1_MoO_8.5_. The enhanced conductivity was correlated with an increase of tetrahedral moieties (*i.e.* (Nb/V/Mo)O_4_) in the palmierite-like layers of the Ba_3_Nb_0.9_V_0.1_MoO_8.5_ crystal structure,^21^ based on changes in the oxygen sublattice upon V-substitution as observed by neutron diffraction.^21^

The further attempts on chemical modification of Ba_3_MoNbO_8.5_-based oxides *via* tailored cationic chemical substitution have not yielded any better oxide-ion conductors compared to the aforementioned Ba_3_Nb_0.9_V_0.1_MoO_8.5_.^23–25,28,30,33^ However, a high oxide-ion and proton conductivity has been discovered in Ba_7_Nb_4_MoO_20_, an anion-deficient, palmierite-perovskite derivative of a 7H polytype of hexagonal perovskite, depicted in Fig. 1(e–h), which exhibits a bulk ionic conductivity of 4.0 mS cm^−1^ at 510 °C.^22^ The compound crystallises in the *P*3̄*m*1 space group and is described as an intergrowth of (12R) perovskite blocks and (9R) palmierite layers along the crystallographic *c*-axis (Fig. 1g).^22,27,32^

There is a significant oxygen occupational disorder along the palmierite-like layers of Ba_7_Nb_4_MoO_20_, which has been described in terms of partially occupied oxygen sites (O1 and O5, Fig. 1h).^22^ These disordered sites result in a situation where mixed polyhedral units (Nb/Mo)O_*x*_ are evidenced in the palmierite-like layers of Ba_7_Nb_4_MoO_20_-based materials (Fig. 1h). The occupation of the O1 site results in tetrahedral (Nb/Mo)O_4_ moieties, while occupation of the O5 site yields octahedral (Nb/Mo)O_6_ units.^18,22^ The O1 and O5 sites have been reported to reorganise upon increasing temperatures, where the O1 site is more occupied than the O5 site.^22^ This reorganisation thus leads to a situation where the tetrahedral units are more prevalent in the palmierite-like layers at high temperatures,^22^ which seems to be a structural trait correlated with increased oxide ion conductivity.

The focus of recent studies on Ba_7_Nb_4_MoO_20_ has been to simultaneously suppress its proton conductivity and increase its oxide-ion conductivity.^18–20,34^ To achieve this, several doped series have been explored, such as Ba_7_Nb_4−*x*_Mo_1+*x*_O_20+*x*/2_,^18^ Ba_7_Ta_4−*x*_Mo_1+*x*_O_20+*x*/2_,^19^ Ba_7_Nb-Cr_*x*_MoO_20+*x*/2_ ^20^ and Ba_7_Nb_4−*x*_W_*x*_MoO_20+*x*/2_.^34^ All of these series have shown enhanced performance with respect to concomitant enhanced oxide-ion conduction and suppressed proton conduction, as well as excellent chemical and electrical stability under reducing atmospheres. The mechanisms of oxide-ion conduction of both the pristine and doped composition have been depicted to proceed *via* a 2D conduction path along the palmierite-like layers.^22^

The conduction properties of Ba_7_Nb_4_MoO_20_-based materials have been hypothesized to improve upon tailored introduction of cations in the palmierite-like layers that preferentially adopt tetrahedral coordination geometry.^22^ For example, V^5+^, Cr^5+^ and Cr^6+^ would be good chemical substitution candidates as they are known to exhibit a preference for tetrahedral coordination in inorganic oxides.^35^ Contrastingly, cations such as W^6+^, Nb^5+^, Mo^6+^ and Ti^4+^ preferentially adopt octahedral coordination geometry.^35^

Given the improvement in ionic conductivity for oxide ions in Ba_3_NbMoO_8.5_ (9R polytype) (Fig. 1d) upon chemical modification with vanadium, it is relevant to determine whether the iso-valent substitution of niobium by vanadium is also feasible for the Ba_7_Nb_4_MoO_20_-based 7H polytype. In case this is feasible, we are interested in the structural modifications brought about by vanadium chemical substitution and whether structural motives conducive to high ionic mobility, such as a propensity for low coordination numbers (*i.e.* CN = 4 instead of 6) for cation sites, in parallel to structural disorder in the oxide anion sub-lattice, are present or become even more pronounced as a function of vanadium content.

Herein, we report the synthesis and characterization of compositions Ba_7_Nb_3.9−*x*_V_*x*_Mo_1.1_O_20.05_ (0 ≤ *x* ≤ 0.4), by substituting V^5+^ in place of Nb^5+^ in Ba_7_Nb_3.9_Mo_1.1_O_20.05_, a composition that is known for both enhanced oxide-ion conduction and suppressed proton conduction.^18^ We employ ^51^V solid-state Nuclear Magnetic Resonance spectroscopy (ss-NMR) to evaluate the local environment of V^5+^ substituents and ^93^Nb NMR to keep track of Nb^5+^ coordination in the series, as well as a combined X-ray and neutron diffraction analysis to study the impact of this chemical modification on O1–O5 oxygen site disorder and occupation of cation sites as a function of temperature. Further insight into the potential conduction pathway and the corresponding energy barriers for oxide-ion migration in the Ba_7_Nb_3.9−*x*_V_*x*_Mo_1.1_O_20.05_ (0 ≤ *x* ≤ 0.4) series is provided by BVSE calculations, showing how vanadium substitution significantly affects the predominant conduction pathway along the palmierite-like layer.

## Experimental

The Ba_7_Nb_3.9−*x*_V_*x*_Mo_1.1_O_20.05_ (0 ≤ *x* ≤ 0.4) series was synthesized using a solid-state reaction approach. Mixtures of stoichiometric amounts of BaCO_3_ (99.95%, Chem-Impex), Nb_2_O_5_ (99.9985%, Puratronic®), V_2_O_5_ (99.99%, Aldrich), and MoO_3_ (≥99.5%, Aldrich) were ground, pelletised and calcined at 1050 °C for 48 h. The calcined pellets were subsequently re-ground, re-pelletised and re-calcined at 1050 °C for 48 h. This subsequent step was repeated one more time to obtain phase pure materials.

Laboratory X-ray powder diffraction (XRD) patterns were collected using a PANanalytical X'Pert Pro X-ray diffractometer equipped with a Cu Kα source (1.54 Å) operated at 45 kV, 40 mA. The XRD patterns were recorded in the range 10° < 2*θ* < 100°. A LaB_6_ NIST standard (NIST660C) was used for both the calibration and performance qualification of the diffractometer.

Neutron powder diffraction (NPD) experiments were performed using PEARL diffractometer of the TU Delft Reactor Institute (the Netherlands).^36^ Approximately 2.0 g of each sample was loaded in a 6 mm diameter can made of a V–Ni null-scattering alloy. The sample can was then placed in a neutron-transparent vacuum box connected to a primary vacuum (∼10^−3^ mbar). Diffraction patterns were measured over approximately 18 h using a wavelength of 1.667 Å, which was selected using the (533) reflection of a single-crystal Ge[511] monochromator. For high-temperature measurements, the temperature was controlled *via* a resistive heater consisting of a vanadium heating element wrapped around the sample can (see details in ESI Fig. S20†).

Structural refinements were performed using the GSAS-II program.^37^ The NPD datasets coupled with the laboratory XRD patterns were used for the refinement of the crystal structure. The instrumental background was fitted with the Chebyshev polynomial, while the unit cell metrics and peak profile parameters were simultaneously refined using the Le Bail intensity extraction.^38^ The Le Bail refinement was subsequently followed by a Rietveld refinement for the determination of the structural model.^39^ For Rietveld structural refinements, fractional coordinates that are not fixed using symmetry were refined independently for all atomic sites alongside their atomic displacement parameters. The fractional occupancy for the barium atomic sites was fixed to its nominal value. The fractional occupancy of M1 and M3 transition metal sites was respectively fixed to 1 and shared between Nb and Mo according to their stoichiometry, whereas the fractional occupancies of both M2 and M4 sites (if observed in the structural model) were constrained to 1 *i.e.* M2 + M4 = 1, and respectively shared between Nb, Mo and V (in the vanadium-doped samples). Likewise, the fractional occupancies of O2, O3 and O4 oxygen sites were respectively fixed to 1 and O1 and O5 were constrained to a nominal value of 2.05 in such a way that [fraction(O(1)) × multiplicity(O(1))] + [fraction(O(5)) × multiplicity(O(5))] = 2.05.

Magic Angle Spinning (MAS) ^51^V and ^93^Nb NMR spectra were recorded with a Bruker Ascend 500 spectrometer (*B*_0_ = 11.7 T) with a NEO console operating at a resonance frequency of 131.557 MHz (^51^V) and 122.291 MHz (^93^Nb), respectively. The samples were spun at desired frequencies in a 3.2 mm rotor. A Bruker two-channel DVT 3.2 mm MAS probe was used for all measurements. The chemical shifts were referenced with respect to V_2_O_5_ (99.99%, Aldrich) with an isotropic chemical shift of −613.2 ppm and NaNbO_3_ (99.997%, Puratronic®) with an isotropic chemical shift of −1130.7 ppm. A solid *π*/2 pulse length of 0.5 μs was used for ^93^Nb NMR data acquisition. For ^51^V measurement, a rotor-synchronised-solid-echo (*π*/2 – *τ* – *π*/2) pulse sequence, with a solid *π*/2 pulse length of 1.5 μs, was used for data acquisition. The acquired ^51^V spectra were simulated using the program DMFit.^40^ We report *δ* as the reduced anisotropy in the Haeberlen Convention, also referred to as axiality of the CSA tensor (*δ*_33_− *δ*_iso_) in the framework of the DMFit program.

The landscape for oxide-ion dynamics was probed using the *softBV*^41^ software. This software utilises a force-field algorithm to compute possible conduction pathways and relative energy barriers for ionic conduction in model crystallographic structures.^42,43^ The computation method implemented in *softBV* is based on the bond-valence sum (BVS) approach as described in ref. 41 and 43. The structural models obtained from Rietveld refinement serve as the input file in which the BVSE landscapes for a probe oxide-ion were calculated. A dense grid of 10 points with a resolution of 0.1 Å was used for the calculations. The diffusion pathways were determined from the regions of low BVSE, which were performed by both visualising the connectivity of the iso-surfaces and examining the calculated pathway segments. The BVSE maps were visualised using VESTA,^44^ and the BVSE model of the migration barrier was plotted from the energy profile of the diffusion pathways.

## Results/discussion

**Fig. 2:**
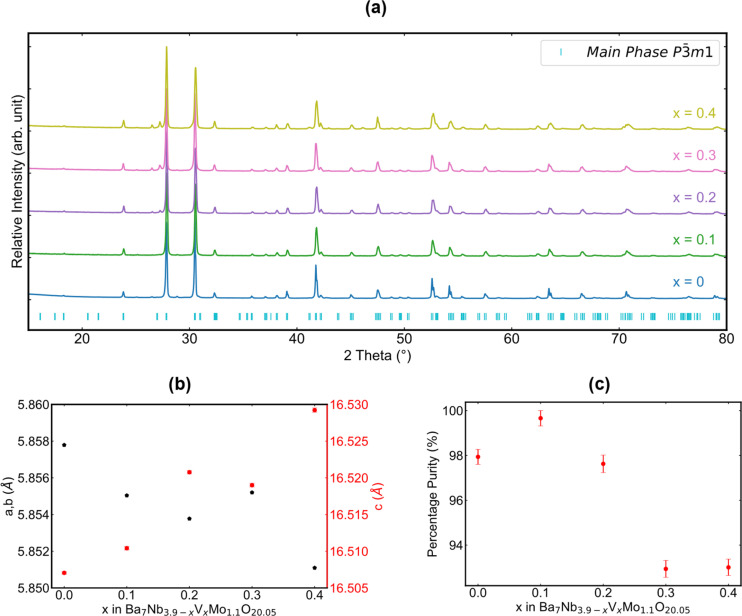
X-ray diffraction analysis of the synthesized Ba_7_Nb_3.9−*x*_V_*x*_Mo_1.1_O_20.05_ series as a function of *x*. (a) XRD patterns, (b) evolution of *a*, *b*, and *c* lattice parameters as a function of *x* and (c) percentage of the main phase. Impurity phases are included in refinement tables and figures in the ESI.†

X-ray diffraction was employed to assess the crystalline phases that resulted from the synthesis of samples in the Ba_7_Nb_3.9−*x*_V_*x*_Mo_1.1_O_20.05_ series. As shown in Fig. 2a, all five patterns exhibit the intended hexagonal-based perovskite as the main phase. Analysis of these patterns provides information about the evolution of lattice parameters in the Ba_7_Nb_3.9−*x*_V_*x*_Mo_1.1_O_20.05_ series, as depicted in Fig. 2b. The lattice parameters of the unit cell are refined using a Le Bail fit^38^ adopting the *P*3̄*m*1 space group. Taking into consideration the Shannon ionic radii of both Nb^5+^ (0.640 Å) and V^5+^ (0.540 Å) in a six-fold coordination or Nb^5+^ (0.480 Å) and V^5+^ (0.355 Å) in four-fold coordination,^45^ the lattice parameters are expected to change towards lower values upon niobium substitution with vanadium. While this expectation is fulfilled for the *a* and *b* lattice parameters, which decrease from 5.858 Å for *x* = 0 to 5.851 Å for *x* = 0.4, an opposite trend is observed in the *c* direction, with an increase of the *c* lattice parameter from 16.509 Å to 16.530 Å. These trends suggest that vanadium incorporation might affect the oxygen distribution between O1 and O5 sites and possibly the metal (Nb, Mo, and V) distribution in M1, M2, M3 and M4 sites, which could conceivably have a non-trivial effect on the lattice volume and shape (*i.e.* non-Vegard-law).

The expansion along the *c* direction indicates a reorganization within the structure to accommodate the isovalent dopant. Upon inspection of the structural model proposed by Garcia-González *et al.*^32^ in Fig. 1h, one can deduce that this expansion could be related to the occupation of additional cation sites (*e.g.* M4) and/or a re-ordering of the oxygen sites within the palmierite-like layer, leading to the repulsion of like ions and the consequent expansion along this direction. Furthermore, this opposing trend for *a* and *c* lattice parameters results in only a minor volume change (∼0.1%) upon vanadium chemical substitution despite the difference in the ionic radii of Nb^5+^ and V^5+^ (Fig. S1†).

Fig. 2c displays the percentage purity of each sample prepared, with respect to the intended phase, as determined by Rietveld refinement (Fig. S2–S10 and Tables S1–S10†). The presence of impurity phases such as BaMoO_4_, Ba_3_Nb_2_O_8_ (and potentially its solid solution with V: Ba_3_Nb_2−*x*_V_*x*_O_8_) and Ba_5_Nb_4_O_15_ was evidenced (Fig. 2a). While a phase purity above 98% was attained for compositions with *x* ≤ 0.2, the formation of side phases was more pronounced for *x* = 0.3 and *x* = 0.4. We also determined that a phase of Ba_7_Nb_3.9−*x*_V_*x*_Mo_1.1_O_20.05_ cannot be obtained for the V end member of the series (*x* = 3.9 – ESI Fig. S11†), which results in the stoichiometric formation of Ba_3_V_2_O_8_ and BaMoO_4_ phases instead. This observation indicates that a high vanadium content destabilizes the 7H polytype and implies a solubility limit for V in the low *x* range. Given this fact, the following questions can be raised about the distribution of vanadium in the substituted samples: is it located in the main or side phases, and in which local coordination environment can we find it?

These two questions, related to both the location of vanadium and origin of structural changes for the 7H polytype upon vanadium modification, will be addressed in the following sections. For the former, we use ^51^V MAS NMR as a local-structure characterization method sensitive to the local coordination of V^5+^. For the latter, we perform an in-depth analysis of combined X-ray and neutron diffraction patterns, leveraging the insight provided by NMR spectroscopy.

### ^51^V MAS NMR spectroscopy

Since NMR spectroscopy is sensitive to interactions between the probed nuclei and their immediate surroundings, it offers advantages over diffraction techniques to gauge the local structure of particular ions in materials, especially when structural disorder may be present.^46,47^^51^V MAS NMR has been used to derive information about the coordination geometry of vanadium in several studies.^48–50^ Given the prominent role of chemical shift anisotropy (CSA) in ^51^V NMR spectra, the evaluation of this parameter plays a key role in the identification of the coordination environment for vanadium.^51^ In particular, Lapina *et al.*^48^ thoroughly compiled CSA tensor parameters of NMR spectra of ^51^V and related them to the local coordination environment of vanadium. In detail, coordination environments with six neighbouring oxide anions in an octahedral shape (*i.e.* V_2_O_5_, Fig. S12†) are related to a reduced chemical shift anisotropy (*δ*) in the range of 300 to 1000 ppm, whereas significantly smaller values are observed for tetrahedral environments (*δ* < 100 ppm).^48^

**Fig. 3:**
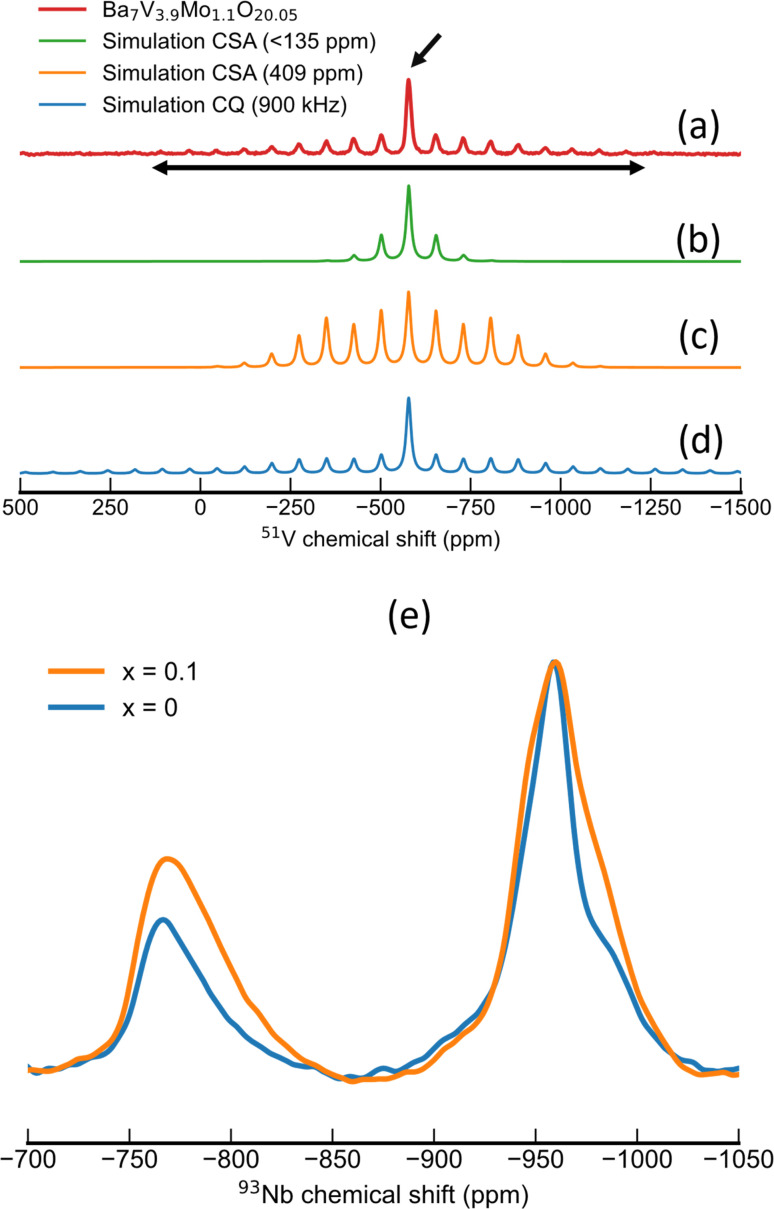
(a–d) ^51^V MAS NMR spectra of Ba_7_Nb_3.8_V_0.1_Mo_1.1_O_20.05_ spun at 10 kHz. Experimental spectrum (a) fitted considering chemical shift anisotropy (b and c) or 1st order quadrupolar interaction (d). The isotropic line is indicated by a slanted, single-headed arrow. The horizontal, double-headed arrow depicts the span of spinning sidebands as a measure for the magnitude of NMR interaction (either chemical shift or quadrupolar coupling). (e) ^93^Nb MAS NMR spectra recorded with a MAS frequency of 38 kHz, with a noticeable intensity increase for tetrahedral NbO_4_ environments upon V-substitution.

Fig. 3a shows the ^51^V MAS NMR spectrum acquired for Ba_7_Nb_3.8_V_0.1_Mo_1.1_O_20.05_, which is characteristic of the series. The isotropic line is marked with an arrow situated at around −577 ppm. This line is flanked by a symmetric spinning sidebands envelope of significantly lower intensity, with a span of around 1000 ppm. Considering the hypothesis that ^51^V NMR spectra are often dominated by the chemical shift anisotropy, the ^51^V NMR spectrum of Ba_7_Nb_3.8_V_0.1_Mo_1.1_O_20.05_ was simulated with only CSA tensor parameters. In this first attempt (Fig. 3b), the distinct intensity between the isotropic peak and spinning sidebands could be reproduced by a small anisotropy, with fitted parameters of *δ* = 135.50 ppm and *η* = 0.97. However, these CSA tensor parameters considerably underestimate the span of the spinning sideband (SSB) envelope from experiments. Conversely, increasing the chemical shift anisotropy in order to fit a broader SSB envelope results in a poor fit of the relative intensities of SSB and the isotropic centre band (Fig. 3c). These observations suggest that the CSA interaction is not a dominant trait in the spectra for ^51^V nuclei in the compounds of interest.

The ^51^V MAS NMR spectrum of Ba_7_Nb_3.8_V_0.1_Mo_1.1_O_20.05_ could be more appropriately simulated considering only the quadrupolar interaction to first order (Fig. 3d). With this interaction, both the relative intensity of the centre band and spinning sidebands, as well as the span of SSBs, could be well reproduced without the need to include the CSA interaction. From the span and shape of the spinning sideband envelope, a *C*_Q_ value of 0.9 MHz with an asymmetry parameter (*η*_Q_) of 0.75 could be extracted from the spectrum with a MAS frequency of 10 kHz. Moreover, the fact that CSA was not needed to simulate these spectra indicates a small magnitude for this interaction, in the order of 135 ppm or smaller. A similar pattern was observed across the series Ba_7_Nb_3.9−*x*_V_*x*_Mo_1.1_O_20.05_ with (0.1 ≤ *x* ≤ 0.4). The corresponding fitted parameters and spectra can be found in the ESI (Table S11 and Fig. S13†).

In light of the empirical correlations from Lapina *et al.*^48^ between the magnitude of the CSA and coordination number for oxy-vanadate and from the observation of small, if not negligible CSA parameters in the ^51^V NMR spectra for the Ba_7_Nb_3.9−*x*_V_*x*_Mo_1.1_O_20.05_ (0.1 ≤ *x* ≤ 0.4) series, we conclude that all V^5+^ cations are located in an isolated tetrahedral environment for the 7H polytype samples investigated. We can thus deduce that V^5+^ is exclusively localised along the palmierite-like layer (M2 site; see Fig. 1h). This conclusion is further corroborated by the observation of a similar pattern of spinning sidebands when analysing the outcome of Ba_3_V_2_O_8_ (Fig. S14†) which features only VO_4_ units in its structure.^52^

Given the fact that V^5+^ exclusively occupies tetrahedral sites, as indicated by the ^51^V NMR spectra discussed above, this preferential coordination is expected to impact the occupation of equivalent cationic sites populated by other metal atoms throughout the structure. As V^5+^ substitutes for Nb^5+^ in the nominal compositions and given the high sensitivity of the latter for local structure investigation *via*^93^Nb NMR, we proceed with the analysis of the Nb^5+^ coordination number.

In the ^93^Nb MAS NMR spectra in Fig. 3e (also in Fig. S18 and S19†), we observe two broad signals which can be assigned to the central transition of ^93^Nb in two distinct local environments. One of these signals has a maximum at −765 ppm, which can be assigned to a NbO_4_ coordination following empirical trends of ^93^Nb chemical shift.^53^ This signal exhibits the following: a rather featureless line shape, slight asymmetry and skewness towards low frequencies. These features can be rationalized in terms of the Czjzek model^54^ and are characteristic of disordered local environments, thus implying a distribution of Nb–O bond lengths or angles for the tetrahedral Nb^5+^ site. The second ^93^Nb NMR signal is located at −958 ppm, which is a chemical shift range typical for octahedral coordination.^55^ Interestingly, this signal presents some finer structures, *e.g.* a significant shoulder at around −980 ppm. This feature is detected in previous reports on ^93^Nb NMR of Ba_7_Nb_4_MoO_20_-based composition.^56,57^ This feature may indicate a well-structured local environment for Nb^5+^ in octahedral environments, as a feature expected of Nb^5+^ occupying for example M1, M3 and/or M4 sites.

From the premise that V exclusively occupies tetrahedral sites, one could expect its substitution for Nb to decrease the relative amount of NbO_4_ moieties. To test this hypothesis, we evaluated the area of ^93^Nb NMR signals assigned to tetrahedral and octahedral Nb^5+^ environments, respectively (Fig. 3e). For the pristine composition (*x* = 0), we observe a tetrahedral-to-octahedral ratio of 25 : 75 for the area of the respective central transitions. Contrastingly, we observe an increase in the relative intensity of the tetrahedral ^93^Nb NMR signal and consequently an increase in the tetrahedral-to-octahedral ratio towards 33 : 67 in the V^5+^ substituted compositions (Fig. S19†). This trend implies that the presence of vanadium in tetrahedral environments influences the local environment of equivalent sites (*i.e.* M2); namely, it appears that the preferential occupation of tetrahedral sites by vanadium influences the oxygen sublattice in such a way that it promotes a tetrahedral coordination also in M2 sites occupied by Nb^5+^. This hypothesis is further evaluated with particular attention to the occupation of O1 and O5 sites along the palmierite layer.

Furthermore, spectral features of the central transition diminished upon vanadium substitution. This is a behaviour often observed^58^ upon both chemical substitution and the consequent increase in structural disorder in adjacent atomic sites, thus supporting the argument of V^5+^ substitution having a long-range effect on the oxygen sublattice.

### Ba_3_Nb_2−*x*_V_*x*_O_8_ side phase

A vanadium-rich side phase could also be detected. The ^51^V chemical shifts of Ba_3_V_2_O_8_ and 7H polytype samples are −601.5 and −577.6 ppm, respectively, which can be well resolved in the ^51^V MAS NMR spectra (Fig. S15†). Despite the similarity in the coordination number, which reflects similar CSA and *C*_Q_ values, the evaluation of the isotropic chemical shift for the Ba_3_V_2_O_8_ and 7H polytype samples still allows for a clear distinction for the local environment of V^5+^ between these two oxide structures. Moreover, the observation of a shoulder at approximately −600 ppm for ^51^V MAS NMR spectra of the 7H polytype samples, whose intensity increases with the substituent concentration (Fig S15b–d†), suggests that Ba_3_V_2_O_8_ (or the isostructural solid-solution Ba_3_Nb_2−*x*_V_*x*_O_8_) is the most probable V-rich side phase—albeit its presence in the sub % concentration as estimated from the spectra in Fig. S15.† We thus further utilize the ^51^V MAS NMR spectra to quantify the amount of Ba_3_Nb_2−*x*_V_*x*_O_8_ impurities (see Table 1 and Ba_3_Nb_2−*x*_V_*x*_O_8_ side phase discussion in the ESI†).

**Table 1:** Vanadium content in the 7H polytype phase and Ba_3_Nb_2−*x*_V_*x*_O_8_ solid solution in the synthesized Ba_7_Nb_3.9−*x*_V_*x*_Mo_1.1_O_20.05_ (0 ≤ *x* ≤ 0.4) series

Nominal composition	Lattice parameters (*a*, *b*, and *c*) of the Ba_3_Nb_2−*x*_V_*x*_O_8_ side phase		*x* in Ba_3_Nb_2−*x*_V_*x*_O_8_ from Vegard's law	% of Ba_3_Nb_2−*x*_V_*x*_O_8_ from Rietveld refinement	Corrected composition XRD	Corrected composition NMR
0	—	—	—	—	—	—
0.1^a^	—	—	—	—	—	—
0.2	5.9032	21.3088	1.0369	3.50	0.16	0.18
0.3	5.9012	21.2897	1.0512	4.80	0.25	0.22
0.4	5.9055	21.3055	1.0201	5.70	0.34	0.37

a The Ba_3_Nb_1.9_V_0.1_O_8_ side phase is not observed for Ba_7_Nb_3.8_V_0.1_Mo_1.1_O_20.05_ within the detection limit.

The resulting actual composition of Ba_3_Nb_2−*x*_V_*x*_O_8_ solid solution is fixed in the structural model of Ba_3_Nb_2−*x*_V_*x*_O_8_ for the further Rietveld refinement of the diffraction patterns. Based on the corrected stoichiometry and knowledge of the local environment of vanadium in the Ba_7_Nb_3.9−*x*_V_*x*_Mo_1.1_O_20.05_ (0 ≤ *x* ≤ 0.4) series, the combined refinement of both the X-ray and neutron diffraction is performed in a self-consistent way to gauge the impact of vanadium doping on the oxygen sub-lattice of the Ba_7_Nb_3.9−*x*_V_*x*_Mo_1.1_O_20.05_ (0 ≤ *x* ≤ 0.4) series.

### Structure refinement from combined X-ray and neutron diffraction

Next, we perform a combined Rietveld refinement for X-ray and neutron diffraction patterns for all five compositions with 0 < *x* < 0.4, in order to analyse the impact of the preferential occupation of tetrahedral sites by vanadium on the global structure of these hexagonal-based perovskite oxides of the 7H polytype. This analysis was performed both at room temperature and at an elevated temperature (527 °C, neutrons only), which is representative of the intermediate temperature range for the operation of SOFCs.

### Base composition

**Fig. 4:**
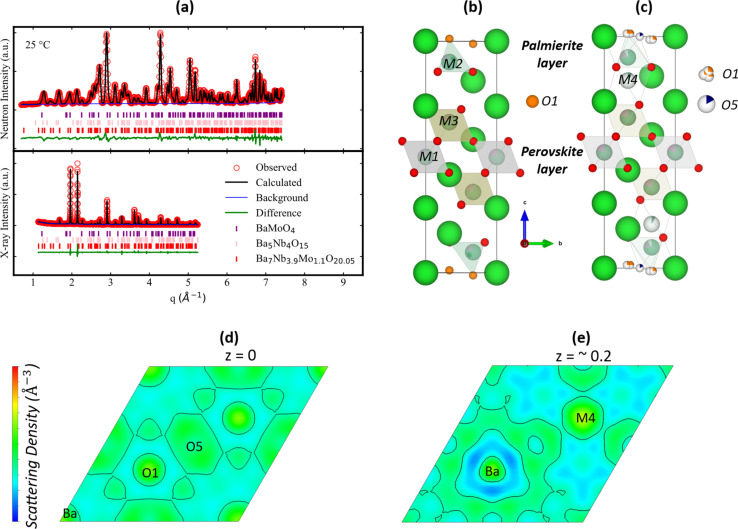
(a) A plot of combined Rietveld refinement of both the X-ray and neutron diffraction patterns of Ba_7_Nb_3.9_Mo_1.1_O_20.05_ at 25 °C (*x* = 0). The crystallographic model (b) proposed by Garcia-González *et al.*^32^ and (c) derived in this work (Table S2†). The different Fourier maps observed in the [001] direction: (d) and (e) maps are calculated from the crystallographic model proposed by Garcia-González *et al.*^32^ These maps evidenced missing scattering densities of both O5 and M4 from the proposed model. The values of *z* for (d) and (e) are 0 and ∼0.2.

Starting with the pristine sample Ba_7_Nb_3.9_Mo_1.1_O_20.05_, a combined neutron and X-ray refinement was performed (Fig. 4a) using the structural model (Fig. 4b) described by Garcia-González *et al.*^32^ It is worth noting that it is hard to establish the site ordering of each cation (Nb^5+^ and Mo^6+^) in Ba_7_Nb_3.9_Mo_1.1_O_20.05_ because of their similar electron density and coherent neutron scattering cross-section^59^ meaning that their relative distribution in the metal sites does not affect the quality of the structural refinements. After fitting the structural model by Garcia-González *et al.*^32^ to the observed patterns, a large thermal displacement parameter (*U*_iso_ = 0.081(8) Å^2^) was observed for the O1 site (Fig. 4b) which was initially at the Wyckoff position 2d (1/3, 2/3, 0.4862(10)). A similar observation has been reported for 7H polytype Ba_7_Nb_4_MoO_20_.^22,32^ Thus, a 3-fold split was applied on the O1 site, which assigns it to Wyckoff position 6i (0.31729, 0.63458, 0.98719), depicted in Fig. 4c. Fitting this to the observed data resulted in a more realistic thermal displacement parameter [0.053(9) Å^2^] for the O1 site. An examination of the neutron difference Fourier map shows two areas of missing scattering density in real space. One of these is at the Wyckoff position 3e (1/2, 0, 0) (Fig. 4d), and it was tagged as O5. The other reveals an additional metal atom, tagged M4, at the Wyckoff position 2d (1/3, 2/3, ∼0.2) (Fig. 4e). The Rietveld refinement of this model resulted in an even better agreement between both the observed and calculated patterns (Fig. 4a). It is worth noting that occupation of O5 and M4 sites has also been reported in Ba_7_Nb_4_MoO_20_ (ref. 22) (Fig. 1h).

**Fig. 5:**
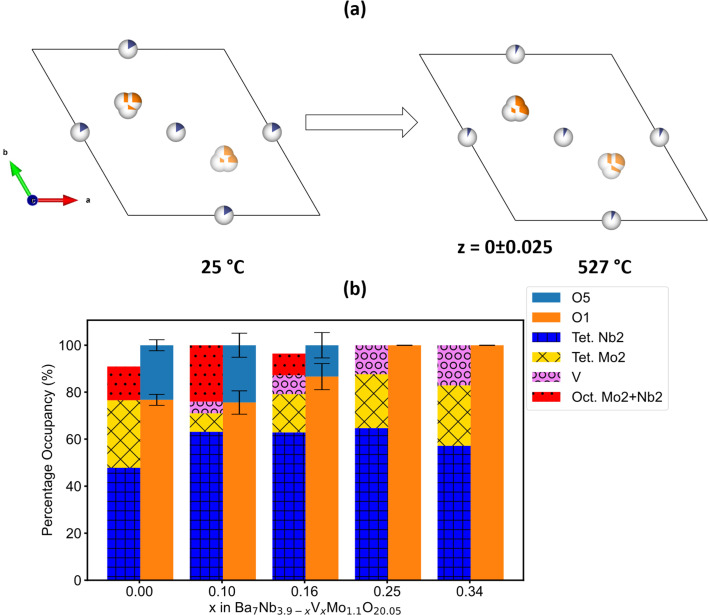
Oxygen and metal-ion distribution in Ba_7_Nb_3.9−*x*_V_*x*_Mo_1.1_O_20.05_ (a) *ab* plane depiction of the thermal reorganisation of oxide-ion site disorder in Ba_7_Nb_3.9_Mo_1.1_O_20.05_. (b) Bar plots depicting the percentage occupancy of oxide-ion disorder and cations in the mixed coordinated M2 site in the Ba_7_Nb_3.9−*x*_V_*x*_Mo_1.1_O_20.05_ series at 25 °C.

From the ^93^Nb ss-NMR observation on the pristine sample, a tetrahedral-to-octahedral ratio of 24.5 : 75.5 was observed for the local environment of Nb. This implies that 24.5% of the nominal stoichiometry of Nb (0.96/3.9 per formula unit) occupies the tetrahedrally coordinated M2 site alongside Mo and the remaining fraction (2.94)—alongside Mo—is distributed over the octahedrally coordinated M1, M2, M3 and M4 sites. This information about the local environment of Nb^5+^ thus results in the splitting of the M2 site into M2-tet and M2-oct, respectively. And this splitting leads to the creation of additional refinement constraints such that the occupation of M2-tet is equal to O1 occupation (Fig. 5b).

In the obtained crystallographic model (Fig. 4c), the distance between M2 and M4 is approximately 1.659 Å and both cationic sites are partially occupied, leading to face-sharing polyhedral units. Also, the distance between the partially occupied O1 and O5 sites is approximately 1.866 Å. The short distance between both the M2–M4 and O1–O5 indicates that they should not be occupied simultaneously. The presence of O1 and O5 sites will lead to mixed coordination polyhedra for the transition d-block metal cation at the M2 site in the palmierite-like layers of Ba_7_Nb_3.9_Mo_1.1_O_20.05_. On an average scale, occupation of O1 will lead to a tetrahedral coordination geometry of the M2 site (*e.g.* M2(O2)_3_O1). Contrastingly, occupation of the O5 site will result in an octahedral coordination geometry for the M2 site cation (*e.g.* M2(O2)_3_(O5)_3_). This observation is analogous to that reported for the local atomic scale of the palmierite derivative of 9R hexagonal perovskite, Ba_3_MoNbO_8.5_, based on data from the neutron pair-distribution function.^60^ From this comparison between the 9R and the present 7H perovskites, a mix of four- and six-fold local coordination environments is expected for M2 cations in the palmierite-like layers of Ba_7_Nb_3.9−*x*_V_*x*_Mo_1.1_O_20.05_.

After the evaluation of the room temperature structure, we proceed with the refinement of the structural model for the neutron diffraction patterns of Ba_7_Nb_3.9_Mo_1.1_O_20.05_ recorded at 527 °C using the same approach. However, it is worth noting that the Nb tetrahedral-to-octahedral ratio and splitting of the M2 site into tetrahedral and octahedral were not considered, as we do not have ^93^Nb NMR data at this temperature. A thermal reorganisation of O1 and O5 sites was observed. This rearrangement led to a situation where O1 is more occupied and O5 is less occupied at 527 °C, when compared to 25 °C (Fig. S16†), resulting effectively in the exclusive tetrahedral coordination for the M2 site at higher temperatures. A similar behaviour has been observed for Ba_7_Nb_4_MoO_20_ by Fop *et al.*^22^ Furthermore, the cationic M2 and M4 sites were also discovered to reorganise upon increasing temperature (Fig. S16†), and a similar feature has been proposed by Fop *et al.* in Ba_7_Nb_4_MoO_20_.^22^

### Vanadium-based compositions

Following the analysis of the base composition, neutron and X-ray diffraction patterns recorded at 25 °C for the vanadium-doped series Ba_7_Nb_3.9−*x*_V_*x*_Mo_1.1_O_20.05_ (0.1 ≤ *x* ≤ 0.4) were analysed in search of a refined structural model. It is worth noting that it is hard to establish the site ordering of V^5+^ in the Ba_7_Nb_3.9−*x*_V_*x*_Mo_1.1_O_20.05_ series with diffraction techniques. The first reason is the fact that the percentage of V^5+^ in each composition of the studied series is so small that refining the fractional occupancies of V^5+^ in the X-ray diffraction model will be unstable even though the electronic density of V^5+^ is distinct from that of either Nb^5+^ or Mo^6+^. Also, vanadium has a small coherent neutron scattering cross-section with a value of 0.0184 barn,^59^ thus, making the Bragg intensities in NPD as well as the Rietveld refinement insensitive to the location of the vanadium nucleus compared to either niobium or molybdenum nuclei in the crystal structure; hence, highlighting the importance of insight from ^51^V MAS NMR for a more in-depth description of the structure.

From the ^51^V NMR parameters, it was observed that vanadium in the Ba_7_Nb_3.9−*x*_V_*x*_Mo_1.1_O_20.05_ series is found in a tetrahedral coordination environment. Based on this observation, the corrected stoichiometry of vanadium (Table 1 and ESI†) in each series was fixed at the M2-tet site of the structural models used in the refinement. The structural models obtained from these refinements are similar to the one derived from pristine Ba_7_Nb_3.9_Mo_1.1_O_20.05_. However, a correlation can be found between the vanadium content and the relative occupation of O1 and O5 sites for the Ba_7_Nb_3.9−*x*_V_*x*_Mo_1.1_O_20.05_ series at 25 °C (Fig. 5b). For samples with *x* = 0.25 and 0.34, the percentage of O1 occupation increased by ∼25% in comparison to the pristine sample with *x* = 0. It is thus apparent that increasing vanadium content increases the tetrahedral M2(O2)_3_O1 units in the average structure of the 7H polytype samples. This observation of O1–O5 rearrangement is analogous to what was reported in the Ba_3_Nb_1−*x*_V_*x*_MoO_8.5_ series of hexagonal perovskites at room temperature.^21^

Furthermore, a thermal reorganization of O1 and O5 sites was observed in the Ba_7_Nb_3.9−*x*_V_*x*_Mo_1.1_O_20.05_ (0 ≤ *x* ≤ 0.4) series at 527 °C (Fig. S16†), where the fractional occupancy of the O5 site is approaching almost zero and O1 site approaching almost 100%. While it is evident that in the pristine Ba_7_Nb_3.9_Mo_1.1_O_20.05_ composition at 527 °C the tetrahedral M2(O2)_3_O1 is the prevalent coordination geometry for cations at the M2 site in the palmierite-like layer, it is interesting to note that this behaviour is observed already at much lower temperatures for the V-based compositions. In other words, the combined analysis of neutron and X-ray diffraction data suggests that introduction of vanadium to the structure alters the oxygen sublattice in a way to mimic the high-temperature structure already at room temperature; hence, reproducing relevant structural features only observed in the high-temperature phase and hence potentially related to ionic conductivity.

### BVSE

The migration pathways and associated energy barriers for oxide-ion conduction in the average crystal structure of several Ba_7_Nb_4_MoO_20_-based hexagonal perovskites^18–20,34^ have been estimated using the Bond Valence Site Energy (BVSE) approach on the *softBV* program.^41–43^ This program presents the resulting BVSE values of the mobile species (oxide-ions) as an energy landscape, which provides information about the connecting local minima, saddle points (identified using fractional coordinate values), and relative energy barriers along possible conduction pathways.^43^ The reliability of this method as a probe for ionic conduction pathways in inorganic conductors is ascertained by its consistency with results obtained from techniques such as molecular dynamics simulations and the maximum entropy method.^61,62^

The conduction of oxide ions in Ba_7_Nb_4_MoO_20_-based materials has been predicted, using the BVSE approach, to proceed predominantly in a two-dimensional fashion involving the average crystallographic oxygen sites in the palmierite-like layers.^18–20,34^ In a bid to correlate the average crystallographic structure of Ba_7_Nb_4_MoO_20_-based hexagonal perovskites with ionic conduction properties, we implemented the BVSE calculation to study the influence of vanadium-doping on the bond-valence site energy landscape for oxide-ion migration in the average crystal structure of the Ba_7_Nb_3.9−*x*_V_*x*_Mo_1.1_O_20.05_ series. Starting with Ba_7_Nb_3.9_Mo_1.1_O_20.05_, a representative of the series, a BVSE landscape for the interaction of probe oxide-ions was calculated using the refined structural model described in Fig. 4c.

**Fig. 6:**
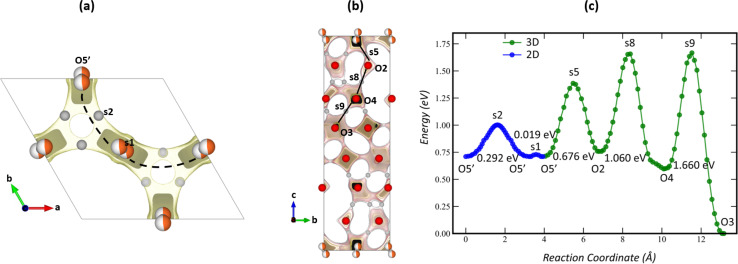
Bond-valence site energy map calculated for Ba_7_Nb_3.9_Mo_1.1_O_20.05_ as seen along the (a) *c*-axis and (b) *a*-axis. (c) Bond-valence energy landscape of Ba_7_Nb_3.9_Mo_1.1_O_20.05_ depicting both two- and three-dimensional conduction pathways.

Fig. 6 depicts the BVSE landscape for oxide-ions and associated conduction pathways obtained for Ba_7_Nb_3.9_Mo_1.1_O_20.05_. In the framework of the BVSE calculation, the disordered O1 and O5 sites in the diffraction-refined model, which are responsible for the mixed coordination of the M2 site (Fig. 4c), are both combined into a partially occupied site tagged O5’ (Fig. 6a). This O5′ site is approximately at the same position as the O5 site from the refined model, only slightly displaced, resulting in double multiplicity (O5′ at (0.486, 0.514, 0.018), Wyckoff 6i; O5 at (1/2, 0, 0), Wyckoff 3e). The O5′ site can be considered an ellipsoidal minimum in the oxygen energy landscape, bridged by a negligible (0.012 eV – Fig. 6c) barrier, denoted as s1 and located at the coordinates of the refined O5.

Interestingly, the BVSE calculation does not identify a local minimum in energy around the coordinates of O1, as would be expected for an appreciably occupied crystallographic position. The reasons for this discrepancy between the diffraction-derived model and the BVSE-derived oxygen positions are not obvious, but the same results have been consistently observed for analogous Ba_7_Nb_4_MoO_20_-based 7H materials in the literature.^18–20,34^ In an attempt, we hypothesize that the BVSE approach is unable to capture the electronic rearrangement of the transition metal in M2 involved in changing its bonding from octahedral (O5) to tetrahedral (O1) coordination, integral to the oxide-ion conduction process; as such, the predominant octahedrally coordinating site (O5) is identified as most stable. The remaining oxygen sites in the structure along with their respective energies and occupancies are listed in Table S13† and exhibit close correspondence between the diffraction- and BVSE-derived structure models.

Furthermore, two-dimensional conduction pathways that involve the diffusion of oxide-ions *via* the partially occupied O5′ sites along the *ab* plane in the palmierite-like layer were evidenced in the computed BVSE map and landscape, consistent with similar studies on Ba_7_Nb_4_MoO_20_-related oxide-ion conductors.^18–20,34^ This 2D-migration pathway has a significantly lower energy barrier than any of the 3D-pathways identified in Fig. 6c, confirming the view of Ba_7_Nb_4_MoO_20_-based 7H materials as 2D conductors. The two-dimensional conduction pathway identified involves two saddle points, tagged s1 and s2 (Fig. 6a and c). The s1 saddle point can be correlated with the local oscillation of oxide-ions in an ellipsoidal minimum, as mentioned above, while the s2 saddle point corresponds to the migration of oxide-ions between different O5′ sites along the O1 position and as such is our main descriptor for oxide-ion mobility in the structure.

**Fig. 7:**
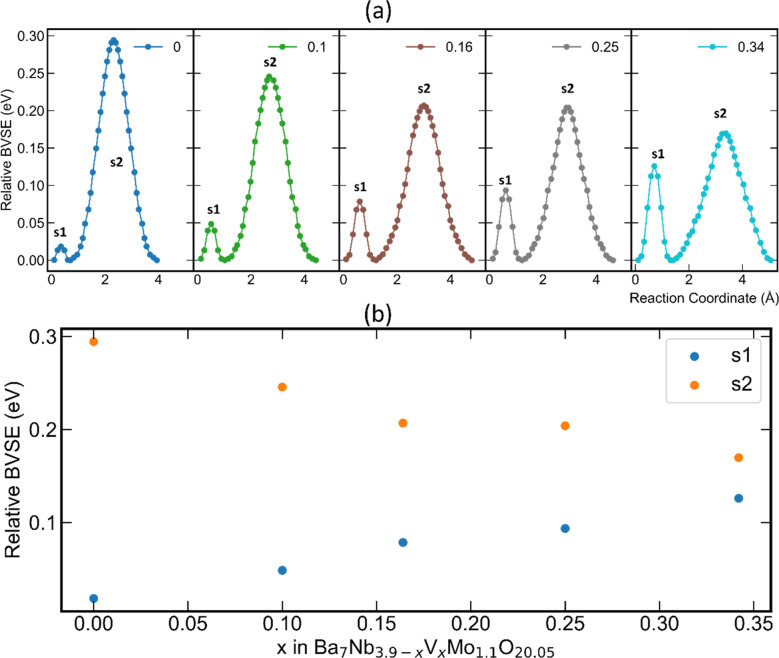
(a) The bond-valence energy landscape for the Ba_7_Nb_3.9−*x*_V_*x*_Mo_1.1_O_20.05_ series illustrating the two saddle points and their respective energy barriers for two-dimensional conduction pathways, and (b) a plot showing the linear variation of the relative bond valence energy of s1 and s2 saddle points as a function of vanadium content in the Ba_7_Nb_3.9−*x*_V_*x*_Mo_1.1_O_20.05_ series.

Fig. 7 depicts the bond-valence energy landscape for the Ba_7_Nb_3.9−*x*_V_*x*_Mo_1.1_O_20.05_ series and the respective energy barriers for s1 and s2 saddle points from the refined structure models as a function of vanadium content. It can be observed that the relative energy barrier of s1 is lower than that of s2 for every member of the series (Fig. 7a and b). Whereas the energy barrier for the s1 saddle point increases linearly with increasing vanadium content in the models, the energy barrier of the s2 saddle point decreases linearly (Fig. 7b). The linear decrease of the energy barrier of the s2 saddle point (Fig. 7b) upon increasing vanadium content suggests that the overall barrier for migration of oxide-ions is lowered by the presence of V^5+^. This observation strongly suggests enhanced oxide-ion mobility in the palmierite-like layer of the Ba_7_Nb_3.9−*x*_V_*x*_Mo_1.1_O_20.05_ series with increasing vanadium content.

Given the fact that a similar bond-valence energy landscape is observed for the structural model reported by Yashima *et al.* for the base composition (*x* = 0) of this 7H polytype,^18^ we expand our BVSE analysis by also considering the potential effect of vanadium occupation on the various metal cation sites of their structural model. In resonance with the trend seen for the relative energy barrier of the s2 saddle in the present structure model, the relative energy barrier of the s2 saddle point for the former model also decreases upon increasing the nominal composition of vanadium at the average M2 crystallographic site (Fig. S17†).

## Conclusion

In this work, we assessed the feasibility of vanadium substitution into the hexagonal-based perovskite oxide of the 7H polytype. We successfully synthesized the Ba_7_Nb_3.9−*x*_V_*x*_Mo_1.1_O_20.05_ series in the 0 ≤ *x* ≤ 0.4 range and thoroughly characterized its average and local structure by a combination of solid-state NMR, X-ray and neutron diffraction.

Based on parameters from ^51^V MAS NMR, we identified a tetrahedral coordination for V^5+^ cations as a chemical substituent for Nb^5+^, indicating its presence in the palmierite-like layers of the structure. Also, it was observed that the preferential occupation of vanadium influences the local environment of adjacent cations in the palmierite layers such that a tetrahedral coordination geometry is enforced on the Nb cation in the M2 site, with ^93^Nb NMR data reinforcing this trend. Combining insight from NMR and XRD, we recognized a solubility limit for vanadium in the low *x* range and identified and quantified V-rich side phases.

Information on the local structures of both vanadium and niobium was essential for the accurate description of the average structure of the main phase and its evolution as a function of V content. A combined X-ray and neutron Rietveld refinement for the undoped sample demonstrated the presence of two distinct oxygen sites along the palmierite-like layer (O1 and O5), whose occupation leads to a mix of four- and six-fold coordination, respectively, for the adjacent M2 cation site. At high temperature (527 °C), a reorganization of oxygen disorder is observed and O1 is found to be more occupied, resulting effectively in a tetrahedral coordination of the M2 site. The same behaviour is observed upon V-substitution, but already at room-temperature, implying that the presence of vanadium impacts the oxygen sub-lattice so as to mimic the high-temperature structure, thereby reproducing at lower temperature structural features conducive to high ionic conductivity.

Our analysis is extended using BVSE calculations to shed light on potential conduction pathways for oxide ions. We found a decreasing energy barrier for O^2−^ migration when vanadium is present in the structure, namely at the M2 site next to the palmierite-like layers, which is the only site able to accommodate a tetrahedral coordination for this substituent. This observation corroborates the hypothesis that vanadium's propensity for a lower coordination number is beneficial for promoting high O^2−^ mobility in this promising class of oxide ion conductors and prompts the study of its effect on the ionic conductivity of this and analogous oxide-ion conductors.

## Data availability

The data supporting this article have been included as part of the ESI.†

## Conflicts of interest

There are no conflicts to declare.

## Supplementary Material

TA-012-D4TA01540A-s001

TA-012-D4TA01540A-s002

TA-012-D4TA01540A-s003

TA-012-D4TA01540A-s004

TA-012-D4TA01540A-s005

TA-012-D4TA01540A-s006

TA-012-D4TA01540A-s007

TA-012-D4TA01540A-s008

TA-012-D4TA01540A-s009

TA-012-D4TA01540A-s010

TA-012-D4TA01540A-s011

TA-012-D4TA01540A-s012
