# Supplementary material for: The role of vanadium substitution in the oxygen sublattice disorder of Ba7Nb4MoO20-based hexagonal perovskite oxide-ion conductors
Source: J Mater Chem A Mater. 2024 Sep 25;12(43):30082–95. doi: 10.1039/d4ta01540a (PMC11484498; doi:10.1039/d4ta01540a)
Supplement: TA-012-D4TA01540A-s001 [file TA-012-D4TA01540A-s001.pdf]

Supplementary Information (SI) for Journal of Materials Chemistry A.
This journal is © The Royal Society of Chemistry 2024

# The Role of Vanadium Substitution in the Oxygen Sublattice Disorder of $Ba_7Nb_4MoO_{20}$-based Hexagonal Perovskite Oxide-Ion Conductors

Abdulkadir Olatunbosun Biffo, Theodosios Famprikis*, and Pedro Braga Groszewicz*

Department of Radiation Science and Technology, Delft University of Technology, Delft 2629JB, Netherlands.

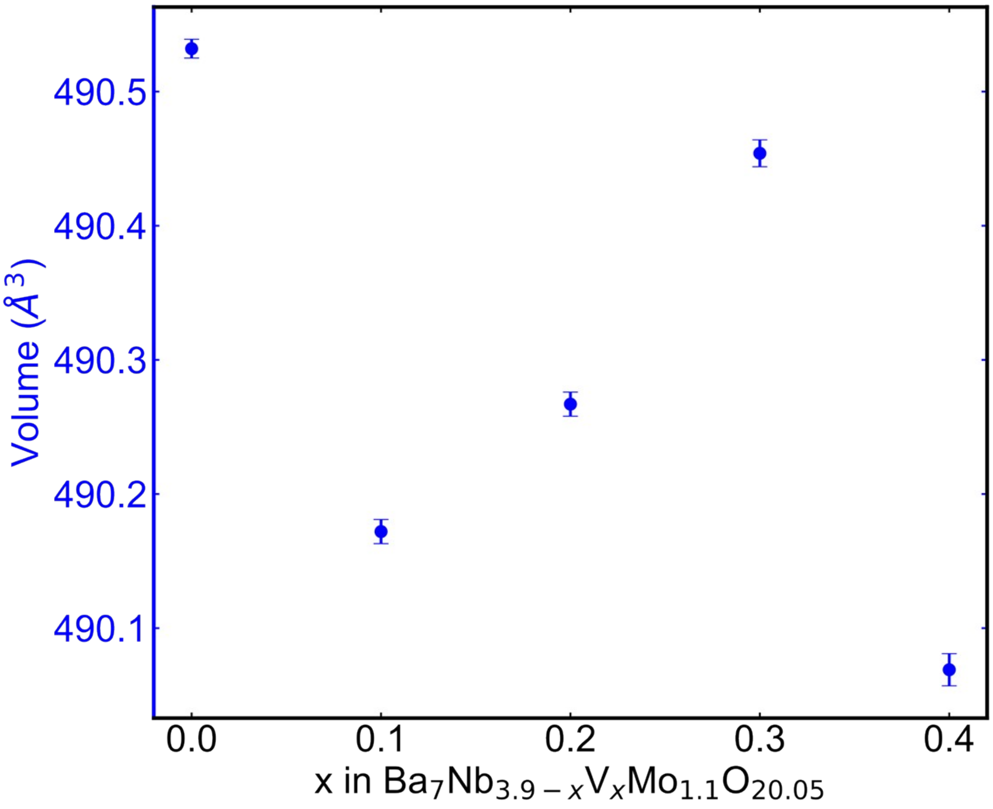


Supporting Figure S1. The evolution of volume of *the synthesized* $Ba_7Nb_{3.9-x}V_xMo_{1.1}O_{20.05}$ *series as a function of x.*

**$Ba_3Nb_{2-x}V_xO_8$ Side Phase:**

The $Ba_3Nb_{2-x}V_xO_8$ solid-solution side phase observed in both the $^{51}V$ MAS NMR spectra (Fig. S15) and from the X-ray diffraction patterns (Fig. 2). The amount of vanadium content in this side phase is quantified from Vegard's law for this solid solution [1] taking into consideration the refined lattice parameters of $Ba_3Nb_{2-x}V_xO_8$ in comparison to the reported lattice parameters of the isostructural end-members $Ba_3Nb_2O_8$ [2], $Ba_3V_2O_8$ [3]. The composition of the $Ba_3Nb_{2-x}V_xO_8$ is then fixed in the further Rietveld refinements of the diffractograms, and the nominal composition of the main phase is corrected to account for vanadium "loss" to the side phase (see Table 1).

Despite the similarity in coordination number —which reflects on the similar CSA and $C_Q$ values— the different isotropic chemical shift for the $Ba_3Nb_{2-x}V_xO_8$ and 7H polytype samples —-601.5 and -577.6 ppm, respectively— still allows for a clear distinction between these phases in $^{51}V$ MAS NMR. We thus further independently quantify the amount of vanadium in $Ba_3Nb_{2-x}V_xO_8$ impurity for each sample through the $^{51}V$ MAS NMR spectra, which is in good agreement with the determination for XRD (Table 1).

Supporting Table S1. Refined atomic parameters from Rietveld refinement of the combined x-ray and neutron diffraction data of $Ba_7Nb_{3.9}Mo_{1.1}O_{20.05}$ at 25 °C. X-ray $R_{wp}$: 7.56%, and Neutron $R_{wp}$: 6.05%.

| | | | | | | |
|---|---|---|---|---|---|---|
| Space Group | $P\bar{3}m1$ | | | | | |
| a (Å) | 5.85802(8) | | alpha (°) | 90 | | |
| b (Å) | =a | | beta(°) | 90 | | |
| c (Å) | | | gamma(°) | 120 | | |
| Volume (Å$^3$) | 490.539(10) | | | | | |
| Label | x | y | z | Frac. Occ. | Uiso | Wyckoff |
| Ba1 | 0 | 0 | 0 | 1 | 0.0077(4) | 1 |
| Ba2 | 0.33333 | 0.66667 | 0.82969(26) | 1 | 0.0077(4) | 2 |
| Ba3 | 0.33333 | 0.66667 | 0.57802(30) | 1 | 0.0077(4) | 2 |
| Ba4 | 0 | 0 | 0.28032(22) | 1 | 0.0077(4) | 2 |
| Nb1 | 0 | 0 | 0.5 | 0.78 | 0.0093(5) | 1 |
| Mo1 | 0 | 0 | 0.5 | 0.22 | 0.0093(5) | 1 |
| Nb2-tet | 0.33333 | 0.66667 | 0.0906(4) | 0.4781 | 0.0093(5) | 2 |
| Mo2-tet | 0.33333 | 0.66667 | 0.0906(4) | 0.2875 | 0.0093(5) | 2 |
| Nb2-oct | 0.33333 | 0.66667 | 0.0906(4) | 0.11 | 0.0093(5) | 2 |
| Mo2-oct | 0.33333 | 0.66667 | 0.0906(4) | 0.0339 | 0.0093(5) | 2 |
| Nb3 | 0.33333 | 0.66667 | 0.3512(3) | 0.9 | 0.0093(5) | 2 |
| Mo3 | 0.33333 | 0.66667 | 0.3512(3) | 0.1 | 0.0093(5) | 2 |
| Nb4 | 0.33333 | 0.66667 | 0.1884(24) | 0.0706 | 0.0093(5) | 2 |
| Mo4 | 0.33333 | 0.66667 | 0.1884(24) | 0.0199 | 0.0093(5) | 2 |
| O1 | 0.34(4) | 0.66(4) | 0.9872(12) | 0.262(7) | 0.070(12) | 6 |
| O2 | 0.1691(10) | 0.8309(10) | 0.1327(4) | 1 | 0.0146(10) | 6 |
| O3 | 0.1645(8) | 0.8355(8) | 0.4332(4) | 1 | 0.0058(9) | 6 |
| O4 | 0.4955(30) | 0.991(6) | 0.29394(25) | 1 | 0.0124(12) | 6 |
| O5 | 0.5 | 0 | 0 | 0.159(13) | 0.070(12) | 3 |
| Impurity Phases | | | | | | |
| $BaMoO_4$ | Space group: I41/a | | | | | |
| a=b (Å) | 5.577(2) | alpha=beta= gamma (°) | 90 | | | |
| c (Å) | 12.866(6) | mass fraction | 0.008(2) | | | |
| volume (Å$^3$) | 400.100(4) | | | | | |
| $Ba_5Nb_4O_{15}$ | Space group: P-3m1 | | | | | |
| a=b (Å) | 5.792(2) | alpha=beta (°) | 90 | | | |
| c (Å) | 11.796(3) | gamma (°) | 120 | | | |
| volume (Å$^3$) | 342.700(1) | | | | | |
| mass fraction | 0.0113(6) | | | | | |

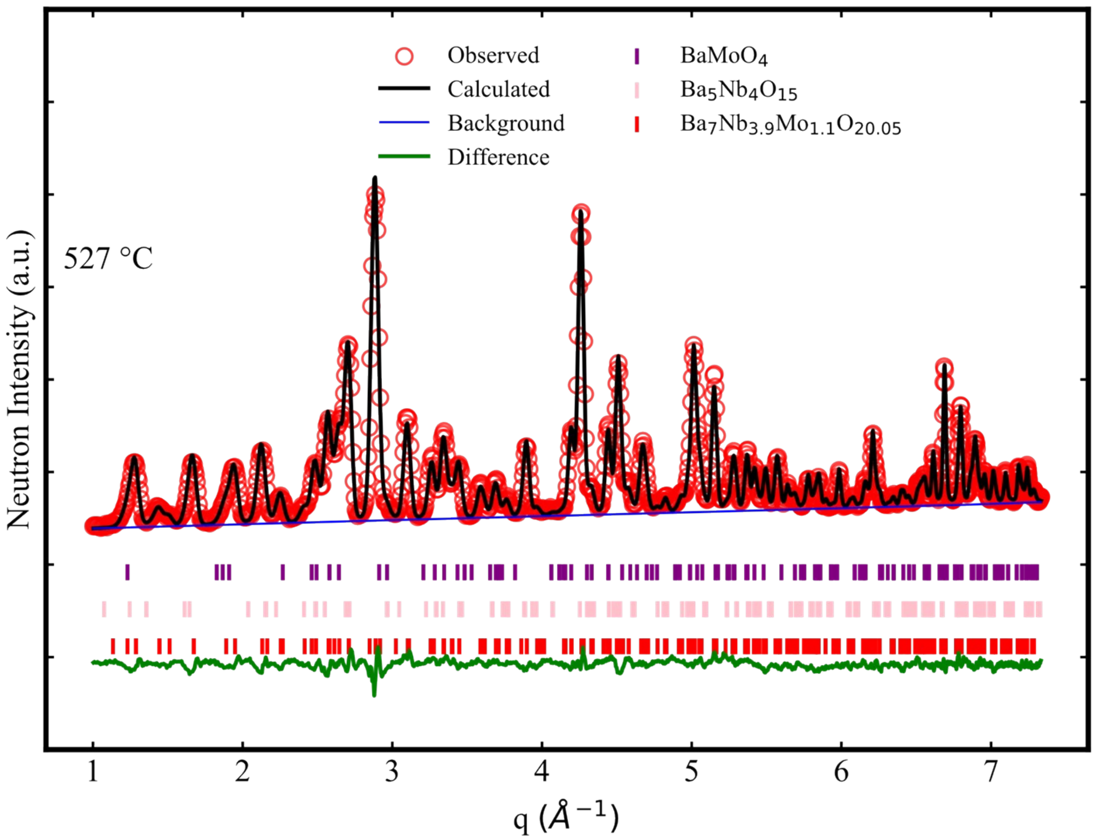


Supporting Figure S2. *A plot of Rietveld refinement of the neutron diffraction pattern of* $Ba_7Nb_{3.9}Mo_{1.1}O_{20.05}$ *at 527 °C (x = 0).*

Supporting Table S2. Refined atomic parameters from Rietveld refinement of neutron diffraction data of $Ba_7Nb_{3.9}Mo_{1.1}O_{20.05}$ at 527 °C. Neutron $R_{wp}$: 4.28%.

| **Space group** | **P-3m1** | | | | | | |
|---|---|---|---|---|---|---|---|
| a (Å) | 5.89685(30) | | alpha (°) | 90 | | | |
| b (Å) | =a | | beta (°) | 90 | | | |
| c (Å) | 16.6222(7) | | gamma (°) | 120 | | | |
| volume ($Å^3$) | 500.565(29) | | | | | | |
| Label | Element | Wyckoff | x | y | z | Fractional Occupancy | Uiso |
| Ba1 | Ba | 1 | 0 | 0 | 0 | 1 | 0.0214(8) |
| Ba2 | Ba | 2 | 0.333333 | 0.666667 | 0.8267(5) | 1 | 0.0214(8) |
| Ba3 | Ba | 2 | 0.333333 | 0.666667 | 0.5778(5) | 1 | 0.0214(8) |
| Ba4 | Ba | 2 | 0 | 0 | 0.2840(5) | 1 | 0.0214(8) |
| Nb1 | Nb | 1 | 0 | 0 | 0.5 | 0.77551 | 0.0172(5) |
| Mo1 | Mo | 1 | 0 | 0 | 0.5 | 0.22449 | 0.0172(5) |
| Nb2 | Nb | 2 | 0.333333 | 0.666667 | 0.0954(4) | 0.77551 | 0.0172(5) |
| Mo2 | Mo | 2 | 0.333333 | 0.666667 | 0.0954(4) | 0.22449 | 0.0172(5) |
| Nb3 | Nb | 2 | 0.333333 | 0.666667 | 0.3524(3) | 0.77551 | 0.0172(5) |
| Mo3 | Mo | 2 | 0.333333 | 0.666667 | 0.3524(3) | 0.22449 | 0.0172(5) |
| O1 | O | 6 | 0.347(7) | 0.652506 | 0.9833(7) | 0.303(4) | 0.066(9) |
| O2 | O | 6 | 0.1704(8) | 0.829572 | 0.13293(29) | 1 | 0.0248(3) |
| O3 | O | 6 | 0.1623(8) | 0.837738 | 0.43397(30) | 1 | 0.0248(3) |
| O4 | O | 6 | 0.4923(25) | 0.984528 | 0.29476(23) | 1 | 0.0248(3) |
| O5 | O | 3 | 0.5 | 0 | 0 | 0.076(9) | 0.0656(9) |

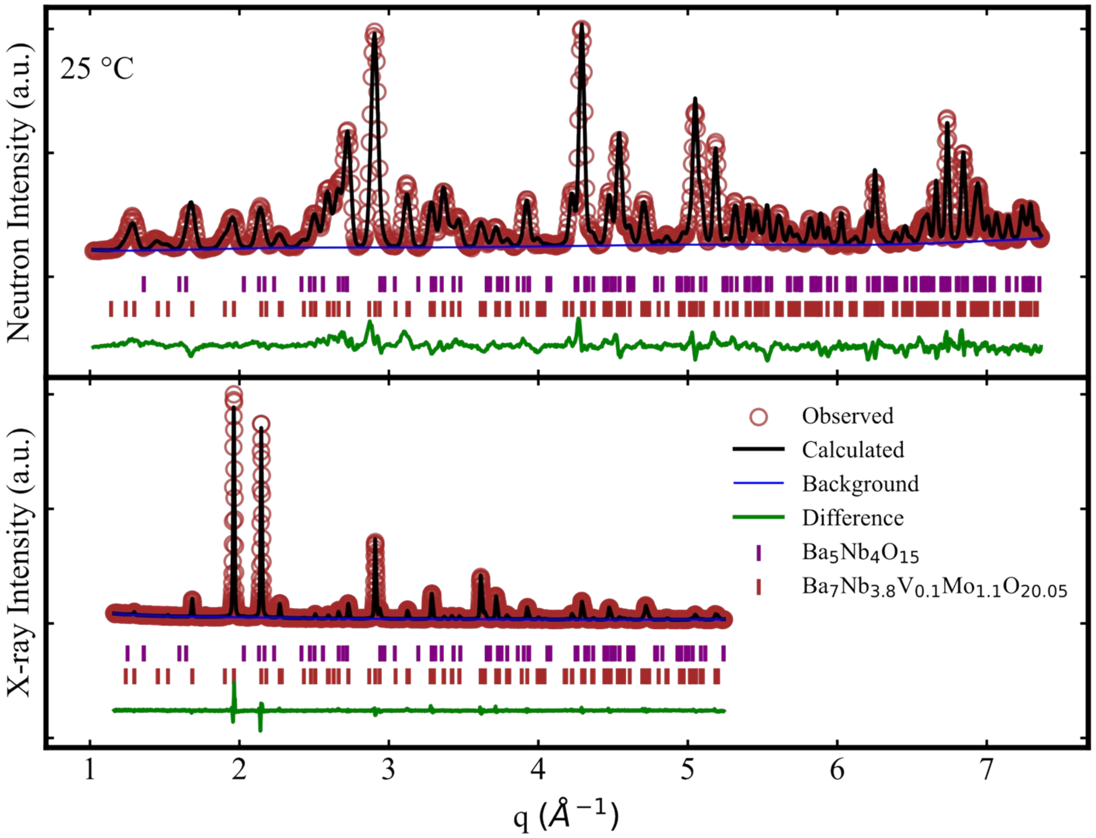


Supporting Figure S3. *A plot of combined Rietveld refinement of both the x-ray and neutron diffraction patterns of* $Ba_7Nb_{3.8}V_{0.1}Mo_{1.1}O_{20.05}$ *at 25 °C (x = 0.1).*

Supporting Table S3. Refined atomic parameters from Rietveld refinement of the combined x-ray and neutron diffraction data of $Ba_7Nb_{3.8}V_{0.1}Mo_{1.1}O_{20.05}$ at 25 °C. X-ray $R_{wp}$: 7.73%, and Neutron $R_{wp}$: 10.22%.

| | | | | | | | |
|---|---|---|---|---|---|---|---|
| Space group | P-3m1 | | | | | | |
| a (Å) | 5.85615(12) | | alpha (°) | 90 | | | |
| b (Å) | =a | | beta (°) | 90 | | | |
| c (Å) | 16.51650(25) | | gamma (°) | 120 | | | |
| volume (Å$^3$) | 490.539(10) | | | | | | |
| Label | Element | x | y | z | Fractional Occupancy | Uiso | Wyckoff |
| Ba1 | Ba | 0 | 0 | 0 | 1 | 0.0055(6) | 1 |
| Ba2 | Ba | 0.33333 | 0.66667 | 0.8297(4) | 1 | 0.0055(6) | 2 |
| Ba3 | Ba | 0.33333 | 0.66667 | 0.5789(4) | 1 | 0.0055(6) | 2 |
| Ba4 | Ba | 0 | 0 | 0.28122(28) | 1 | 0.0055(6) | 2 |
| Nb1 | Nb | 0 | 0 | 0.5 | 0.7755 | 0.0075(10) | 1 |
| Mo1 | Mo | 0 | 0 | 0.5 | 0.2245 | 0.0075(10) | 1 |
| Nb2-tet | Nb | 0.33333 | 0.66667 | 0.0927(5) | 0.6309 | 0.0075(10) | 2 |
| Nb2-oct | Nb | 0.33333 | 0.66667 | 0.0927(5) | 0.1057 | 0.0075(10) | 2 |
| Mo2-tet | Mo | 0.33333 | 0.66667 | 0.0927(5) | 0.0788 | 0.0075(10) | 2 |
| V | V | 0.33333 | 0.66667 | 0.0927(5) | 0.05 | 0.0075(10) | 2 |
| Mo2-oct | Mo | 0.33333 | 0.66667 | 0.0927(5) | 0.1345 | 0.0075(10) | 2 |
| Nb3 | Nb | 0.33333 | 0.66667 | 0.3507(6) | 0.7755 | 0.0075(10) | 2 |
| Mo3 | Mo | 0.33333 | 0.66667 | 0.3507(6) | 0.2245 | 0.0075(10) | 2 |
| O1 | O | 0.355(9) | 0.645(9) | 0.9858(18) | 0.258(13) | 0.005(15) | 6 |
| O2 | O | 0.1709(19) | 0.8291(19) | 0.1323(10) | 1 | 0.0076(13) | 6 |
| O3 | O | 0.1665(23) | 0.8335(23) | 0.4344(10) | 1 | 0.0076(13) | 6 |
| O4 | O | 0.498(7) | 0.995(15) | 0.2957(8) | 1 | 0.0076(13) | 6 |
| O5 | O | 0 | 0.5 | 0 | 0.167(25) | 0.005(15) | 3 |
| Impurity Phases | | | | | | | |
| $BaMoO_4$ | Space group: I41/a | | | | | | |
| a=b (Å) | 5.576(3) | alpha=beta= gamma (°) | | 90 | | | |
| c (Å) | 12.889(7) | mass fraction | | | | 0.0037(5) | |
| volume (Å$^3$) | | 400.800(4) | | | | | |
| $Ba_3V_xNb_{2-x}O8$ | | Space group: R-3m1 | | | | | |
| a=b (Å) | 5.904(2) | alpha=beta (°) | | 90 | | | |
| c (Å) | 21.313(6) | gamma (°) | | 120 | | | |
| volume (Å$^3$) | | 643.400(3) | | | | | |

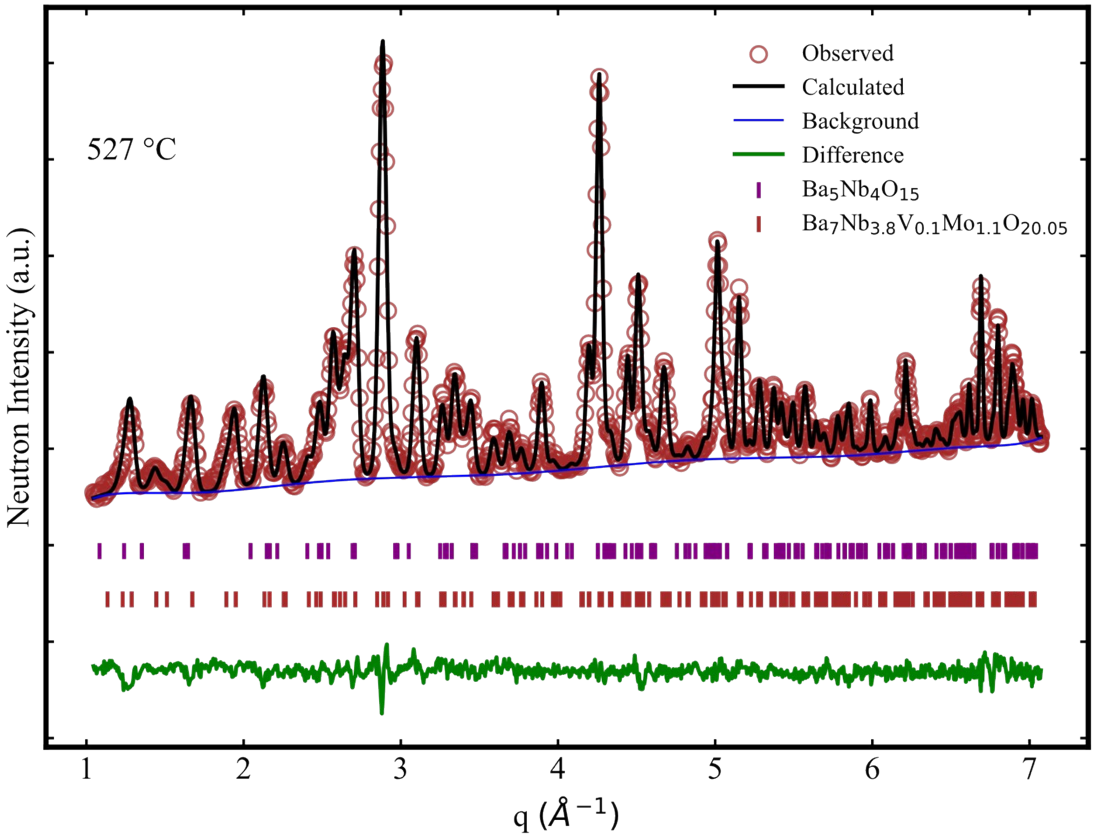


Supporting Figure S4. *A plot of Rietveld refinement of neutron diffraction pattern of $Ba_7Nb_{3.8}V_{0.1}Mo_{1.1}O_{20.05}$ at 527 °C (x = 0.1).*

Supporting Table S4. Refined atomic parameters from Rietveld refinement of neutron diffraction data of $Ba_7Nb_{3.8}V_{0.1}Mo_{1.1}O_{20.05}$ at 527 °C. Neutron $R_{wp}$: 5.05%.

| **Space Group** | **P-3m1** | | | | | | |
|---|---|---|---|---|---|---|---|
| a (Å) | 5.8942(4) | alpha (°) | 90 | | | | |
| b (Å) | =a | beta (°) | 90 | | | | |
| c (Å) | 16.6192(10) | gamma (°) | 120 | | | | |
| volume (Å$^3$) | 500.03(4) | | | | | | |
| Label | Element | Wyckoff | x | y | z | Fractional Occupancy | Uiso |
| Ba1 | Ba | 1 | 0 | 0 | 0 | 1 | 0.0214(11) |
| Ba2 | Ba | 2 | 0.333333 | 0.666667 | 0.8245(8) | 1 | 0.0214 |
| Ba3 | Ba | 2 | 0.333333 | 0.666667 | 0.5749(8) | 1 | 0.0214 |
| Ba4 | Ba | 2 | 0 | 0 | 0.2836(8) | 1 | 0.0214 |
| Nb1 | Nb | 1 | 0 | 0 | 0.5 | 0.77551 | 0.0155(9) |
| Mo1 | Mo | 1 | 0 | 0 | 0.5 | 0.22449 | 0.0155(9) |
| Nb2 | Nb | 2 | 0.333333 | 0.666667 | 0.0927(7) | 0.7336 | 0.0155(9) |
| Mo2 | Nb | 2 | 0.333333 | 0.666667 | 0.0927(7) | 0.21237 | 0.0155(9) |
| V | V | 2 | 0.333333 | 0.666667 | 0.092692 | 0.05 | 0.0155(9) |
| Nb3 | Nb | 2 | 0.333333 | 0.666667 | 0.3490(5) | 0.7755 | 0.0155(9) |
| Mo3 | Mo | 2 | 0.333333 | 0.666667 | 0.3490(5) | 0.2245 | 0.0155(9) |
| Nb4 | Nb | 2 | 0.333333 | 0.666667 | 0.05(16) | 0.0031(1) | 0.0155(9) |
| Mo4 | Mo | 2 | 0.333333 | 0.666667 | 0.05(16) | 0.0009(1) | 0.0155(9) |
| O1 | O | 6 | 0.355(4) | 0.644779 | 0.9879(8) | 0.338(6) | 0.047(9) |
| O2 | O | 6 | 0.1727(12) | 0.827345 | 0.1314(4) | 1 | 0.0257(4) |
| O3 | O | 6 | 0.1620(10) | 0.837974 | 0.4331(5) | 1 | 0.0257(4) |
| O4 | O | 6 | 0.493(3) | 0.986442 | 0.2940(3) | 1 | 0.0257(4) |
| O5 | O | 3 | 0.5 | 0 | 0 | 0.007(12) | 0.0466(9) |

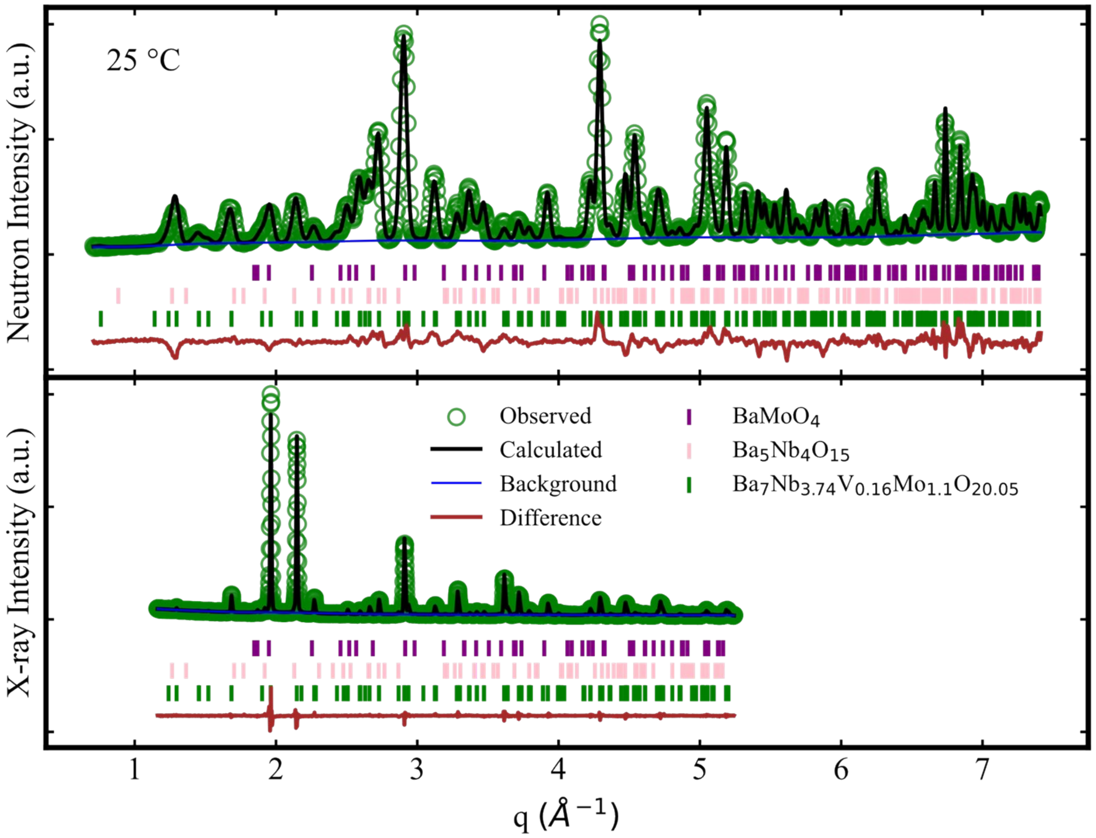


Supporting Figure S5. *A plot of combined Rietveld refinement of both the x-ray and neutron diffraction patterns of Ba7*$Nb_{3.74}V_{0.16}Mo_{1.1}O_{20.05}$ *at 25 °C (x = 0.16).*

Supporting Table S5. Refined atomic parameters from Rietveld refinement of the combined x-ray and neutron diffraction data of $Ba_7Nb_{3.74}V_{0.16}Mo_{1.1}O_{20.05}$ at 25 °C. X-ray $R_{wp}$: 8.63%, and Neutron $R_{wp}$: 11.207%.

| | | | | | | | |
|---|---|---|---|---|---|---|---|
| Space | group: | P-3m1 | | | | | |
| a (Å) | 5.85384 | alpha (°) | | 90 | | | |
| b (Å) | =a | beta (°) | | 90 | | | |
| c (Å) | 16.51747(28) | gamma (°) | | 120 | | | |
| volume (Å$^3$) | 490.180(11) | | | | | | |
| Label | Element | x | y | z | Fractional Occupancy | Uiso | Wyckoff |
| Ba1 | Ba2+ | 0 | 0 | 0 | 1 | 0.0140(8) | 1 |
| Ba2 | Ba2+ | 0.33333 | 0.66667 | 0.8276(5) | 1 | 0.0140(8) | 2 |
| Ba3 | Ba2+ | 0.33333 | 0.66667 | 0.5754(5) | 1 | 0.0140(8) | 2 |
| Ba4 | Ba2+ | 0 | 0 | 0.2799(3) | 1 | 0.0140(8) | 2 |
| Nb1 | Nb5+ | 0 | 0 | 0.5 | 0.2273 | 0.0070(12) | 1 |
| Mo1 | Mo6+ | 0 | 0 | 0.5 | 0.7727 | 0.0070(12) | 1 |
| Nb2-tet | Nb5+ | 0.33333 | 0.66667 | 0.0924(6) | 0.6409 | 0.0070(12) | 2 |
| Nb2-oct | Nb5+ | 0.33333 | 0.66667 | 0.0924(6) | 0.042 | 0.0070(12) | 2 |
| Mo2-tet | Mo6+ | 0.33333 | 0.66667 | 0.0924(6) | 0.166 | 0.0070(12) | 2 |
| Mo2-oct | Mo6+ | 0.33333 | 0.66667 | 0.0924(6) | 0.052 | 0.0070(12) | 2 |
| V | V5+ | 0.33333 | 0.66667 | 0.0924(6) | 0.0819 | 0.0070(12) | 2 |
| Nb3 | Nb5+ | 0.33333 | 0.66667 | 0.3477(7) | 0.7727 | 0.0070(12) | 2 |
| Mo3 | Mo6+ | 0.33333 | 0.66667 | 0.3477(7) | 0.2273 | 0.0070(12) | 2 |
| Nb4 | Nb5+ | 0.33333 | 0.66667 | 0.169(11) | 0.028 | 0.0070(12) | 2 |
| Mo4 | Mo6+ | 0.33333 | 0.66667 | 0.169(11) | 0.0082 | 0.0070(12) | 2 |
| O1 | O2- | 0.361(6) | 0.639(6) | 0.9889(21) | 0.296(14) | 0.015(16) | 6 |
| O2 | O2- | 0.1730(20) | 0.8270(20) | 0.1314(9) | 1 | 0.0113(12) | 6 |
| O3 | O2- | 0.1652(27) | 0.8348(27) | 0.4317(10) | 1 | 0.0113(12) | 6 |
| O4 | O2- | 0.485(7) | 0.969(15) | 0.2952(7) | 1 | 0.0113(12) | 6 |
| O5 | O2- | 0.5 | 0 | 0 | 0.091(28) | 0.015(16) | 3 |
| Impurity Phases | | | | | | | |
| $BaMoO_4$ | | Space group: I41/a | | | | | |
| a=b (Å) | 5.576(3) | alpha=beta= gamma (°) | | 90 | | | |
| c (Å) | 12.889(7) | mass fraction | | 0.0037(5) | | | |
| volume (Å$^3$) | | 400.800(4) | | | | | |
| $Ba_3V_xNb_{2-x}O8$ | | Space group: R-3m1 | | | | | |
| a=b (Å) | 5.904(2) | alpha=beta (°) | | | 90 | | |
| c (Å) | 21.313(6) | gamma (°) | | | | 120 | |
| volume (Å$^3$) | | 643.400(3) | | | | | |
| mass fraction | | 0.0075(7) | | | | | |

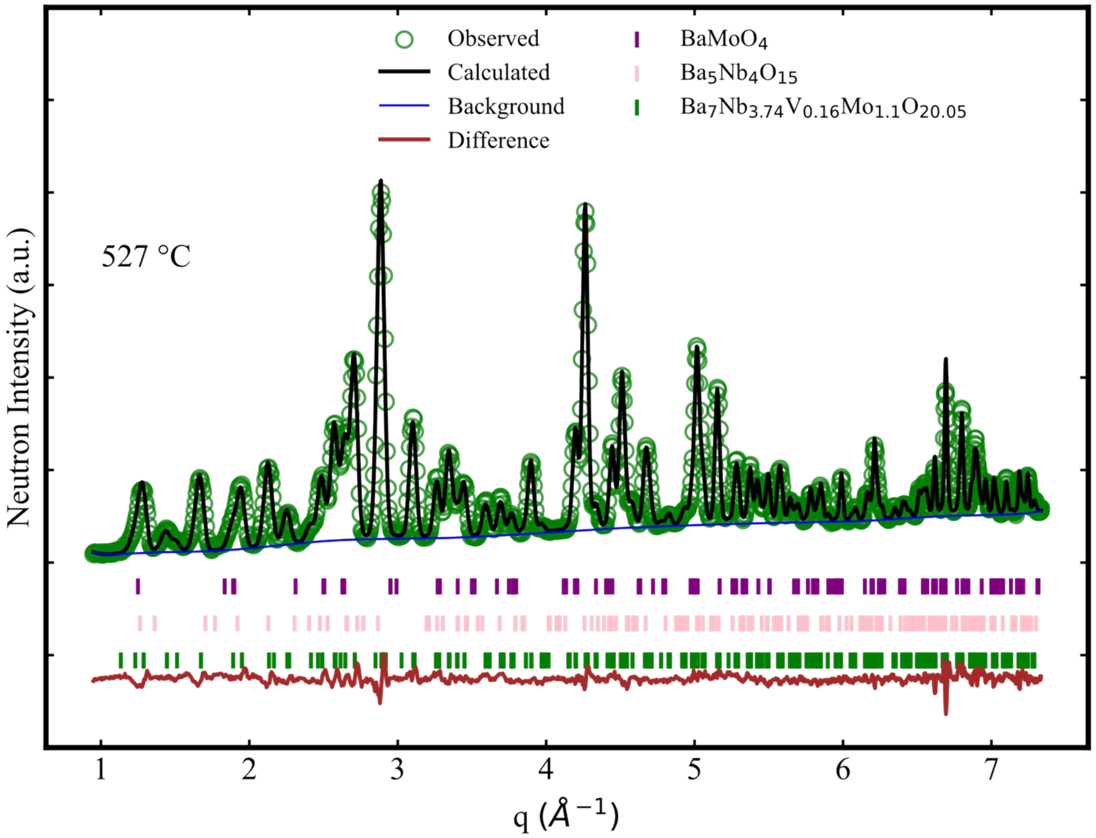


Supporting Figure S6. *A plot of Rietveld refinement of neutron diffraction patterns of* $Ba_7Nb_{3.74}V_{0.16}Mo_{1.1}O_{20.05}$ *at 527 °C (x = 0.16).*

Supporting Table S6. Refined atomic parameters from Rietveld refinement of neutron diffraction data of $Ba_7Nb_{3.74}V_{0.16}Mo_{1.1}O_{20.05}$ at 527 °C. Neutron $R_{wp}$: 6.08%.

| **Space Group** | **P-3m1** | | | | | | |
|---|---|---|---|---|---|---|---|
| a (Å) | 5.8930(3) | alpha (°) | | 90 | | | |
| b (Å) | =a | beta (°) | | 90 | | | |
| c (Å) | 16.6165(7) | gamma (°) | | 120 | | | |
| volume ($Å^3$) | 499.734(31) | | | | | | |
| label | Element | Wyckoff | x | y | z | Fractional Occupancy | Uiso |
| Ba1 | Ba+2 | 1 | 0 | 0 | 0 | 1 | 0.0233(9) |
| Ba2 | Ba+2 | 2 | 0.33333 | 0.66667 | 0.8251(6) | 1 | 0.0233(9) |
| Ba3 | Ba+2 | 2 | 0.33333 | 0.66667 | 0.5753(6) | 1 | 0.0233(9) |
| Ba4 | Ba+2 | 2 | 0 | 0 | 0.2839(5) | 1 | 0.0233(9) |
| Nb1 | Nb+5 | 1 | 0 | 0 | 0.5 | 0.772539 | 0.0145(5) |
| Mo1 | Mo+6 | 1 | 0 | 0 | 0.5 | 0.227461 | 0.0145(5) |
| Nb2 | Nb+5 | 2 | 0.33333 | 0.66667 | 0.0958(4) | 0.709191 | 0.0145(5) |
| Mo2 | Mo+6 | 2 | 0.33333 | 0.66667 | 0.0958(4) | 0.208809 | 0.0145(5) |
| V | V | 2 | 0.333333 | 0.666667 | 0.095762 | 0.082 | 0.0145(5) |
| Nb3 | Nb+5 | 2 | 0.33333 | 0.66667 | 0.3509(3) | 0.772539 | 0.0145(5) |
| Mo3 | Mo+6 | 2 | 0.33333 | 0.66667 | 0.3509(3) | 0.227461 | 0.0145(5) |
| O1 | O-2 | 6 | 0.3608(23) | 0.639227 | 0.9875(7) | 0.330(5) | 0.046(6) |
| O2 | O-2 | 6 | 0.1707(8) | 0.829322 | 0.13065(28) | 1 | 0.0253(4) |
| O3 | O-2 | 6 | 0.1632(8) | 0.836796 | 0.43243(32) | 1 | 0.0253(4) |
| O4 | O-2 | 6 | 0.4939(25) | 0.987737 | 0.29479(24) | 1 | 0.0253(4) |
| O5 | O-2 | 3 | 0.5 | 0 | 0 | 0.023(10) | 0.046 (6) |

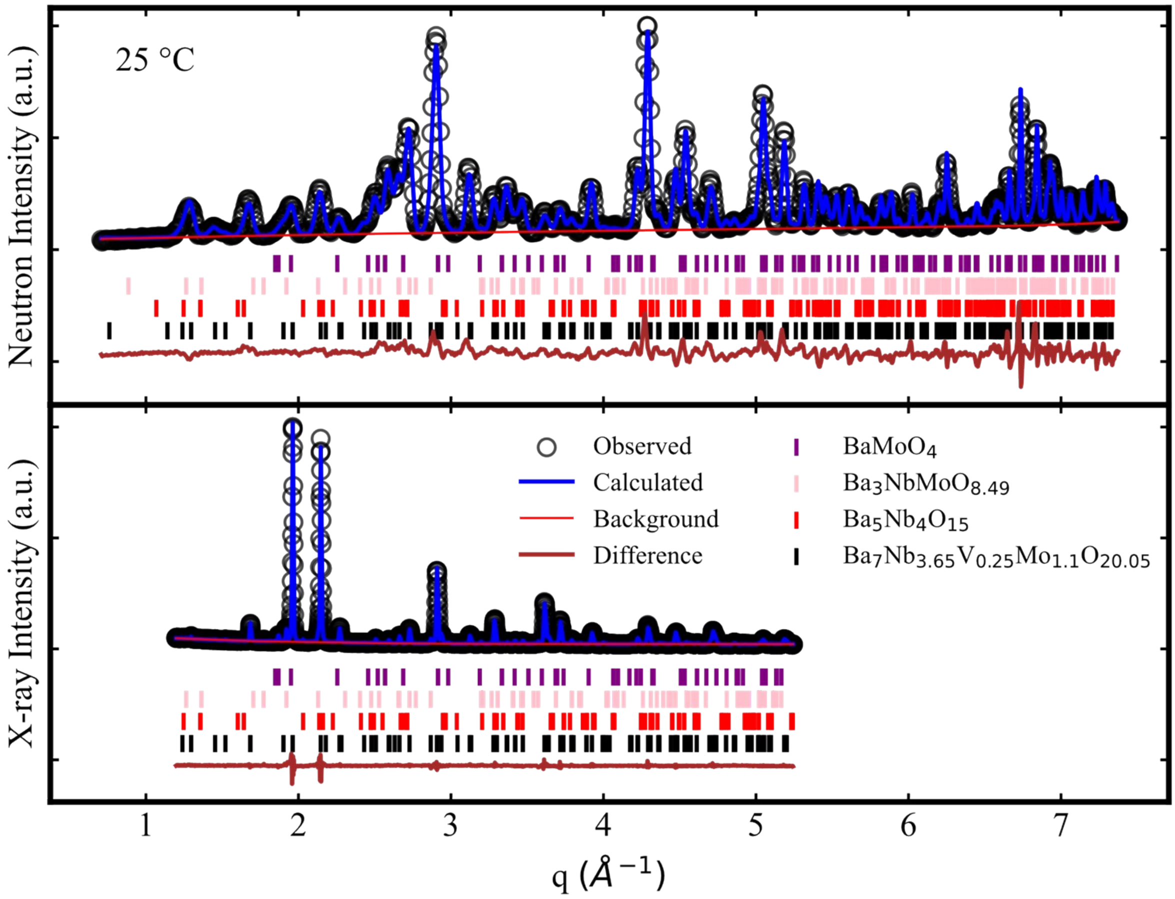


Supporting Figure S7. *A plot of combined Rietveld refinement of both the x-ray and neutron diffraction patterns of* $Ba_7Nb_{3.65}V_{0.25}Mo_{1.1}O_{20.05}$ *at 25 °C (x = 0.25).*

Supporting Table S7. Refined atomic parameters from Rietveld refinement of the combined x-ray and neutron diffraction data of $Ba_7Nb_{3.65}V_{0.25}Mo_{1.1}O_{20.05}$ at 25 °C. X-ray $R_{wp}$: 7.56%, and Neutron $R_{wp}$: 11.54%.

| Space group | | P-3m1 | | | | | |
|---|---|---|---|---|---|---|---|
| a (Å) | 5.85466(13) | | alpha (°) | 90 | | | |
| b (Å) | =a | | beta (°) | 90 | | | |
| c (Å) | 16.51800(27) | | gamma (°) | 120 | | | |
| volume (Å$^3$) | 490.334(11) | | | | | | |
| Label | Element | x | y | z | Fractional Occupancy | Uiso | Wyckoff |
| Ba1 | Ba | 0 | 0 | 0 | 1 | 0.0099(7) | 1 |
| Ba2 | Ba | 0.33333 | 0.66667 | 0.8290(4) | 1 | 0.0099(7) | 2 |
| Ba3 | Ba | 0.33333 | 0.66667 | 0.5754(4) | 1 | 0.0099(7) | 2 |
| Ba4 | Ba | 0 | 0 | 0.27999(30) | 1 | 0.0099(7) | 2 |
| Nb1 | Nb | 0 | 0 | 0.5 | 0.7684 | 0.0030(11) | 1 |
| Mo1 | Mo | 0 | 0 | 0.5 | 0.2316 | 0.0030(11) | 1 |
| Nb2-tet | Nb | 0.33333 | 0.66667 | 0.0944(6) | 0.6544 | 0.0030(11) | 2 |
| Mo2-tet | Mo | 0.33333 | 0.66667 | 0.0944(6) | 0.2322 | 0.0030(11) | 2 |
| V | V | 0.33333 | 0.66667 | 0.0944(6) | 0.1248 | 0.0030(11) | 2 |
| Nb3 | Nb | 0.33333 | 0.66667 | 0.3489(7) | 0.7864 | 0.0030(11) | 2 |
| Mo3 | Mo | 0.33333 | 0.66667 | 0.3489(7) | 0.202 | 0.0030(11) | 2 |
| O1 | O | 0.352(9) | 0.648(9) | 0.9850(14) | 0.341667 | 0.015(16) | 6 |
| O2 | O | 0.1665(20) | 0.8335(20) | 0.1318(9) | 1 | 0.0103(12) | 6 |
| O3 | O | 0.1702(26) | 0.8298(26) | 0.4324(10) | 1 | 0.0103(12) | 6 |
| O4 | O | 0.489(8) | 0.978(16) | 0.2953(8) | 1 | 0.0103(12) | 6 |

Impurity Phases

| $BaMoO_4$ | | Space group: I41/a | |
|---|---|---|---|
| a=b (Å) | 5.572(2) | alpha=beta= gamma (°) | 90 |
| c (Å) | 12.879(3) | mass fraction | 0.0109(5) |
| volume (Å$^3$) | 399.800(2) | | |
| $Ba_3V_xNb_{2-x}O8$ | | Space group: R-3m1 | |
| a=b (Å) | 5.901(1) | alpha=beta (°) | 90 |
| c (Å) | 21.291(3) | gamma (°) | 120 |
| volume (Å$^3$) | | 642.12(1) | |
| mass fraction | | 0.050(1) | |
| $Ba_5Nb_4O_{15}$ | | Space group: P-3m1 | |
| a=b (Å) | 5.810(2) | alpha=beta (°) | 90 |
| c (Å) | 11.760(3) | gamma (°) | 120 |
| volume (Å$^3$) | | 344.169(1) | |
| mass fraction | | 0.00022(5) | |

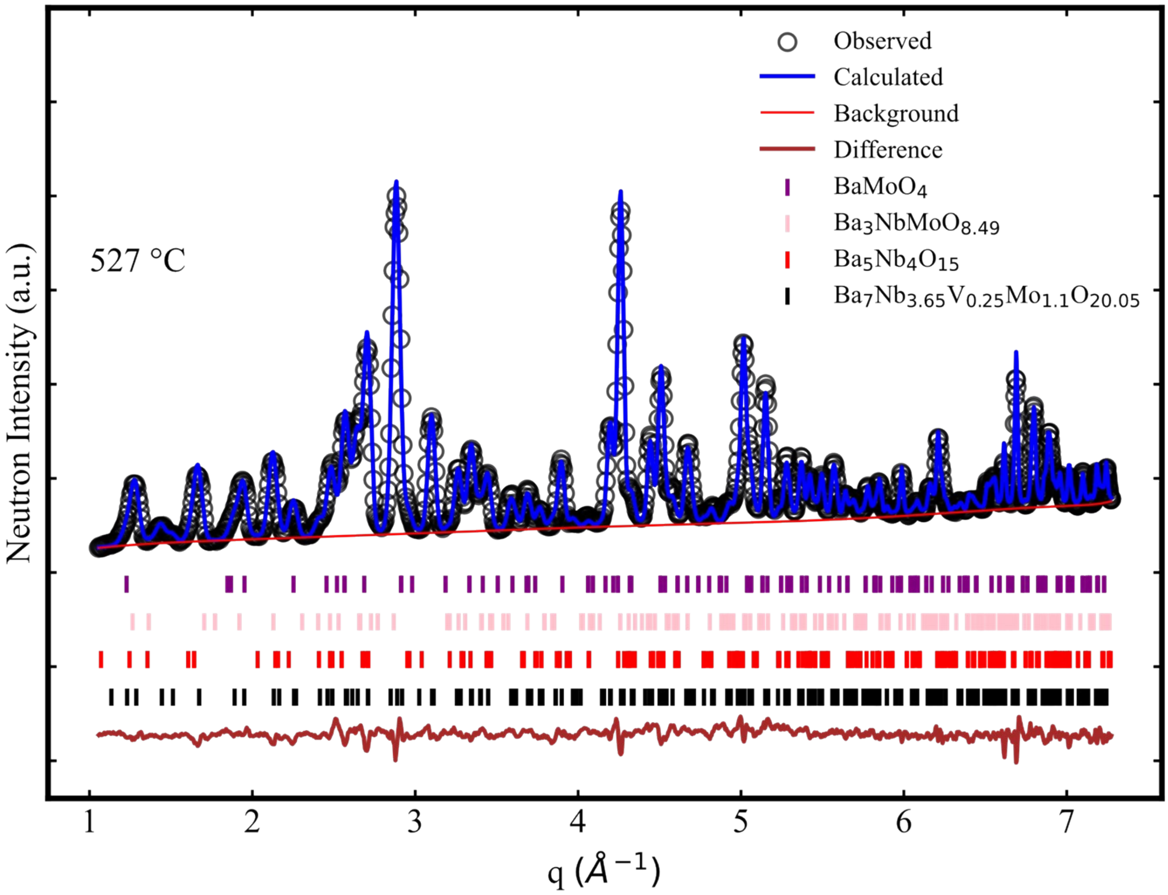


Supporting Figure S8. *A plot of Rietveld refinement of neutron diffraction patterns of* $Ba_7Nb_{3.65}V_{0.25}Mo_{1.1}O_{20.05}$ *at 527 °C (x = 0.25).*

Supporting Table S8. Refined atomic parameters from Rietveld refinement of neutron diffraction data of $Ba_7Nb_{3.65}V_{0.25}Mo_{1.1}O_{20.05}$ at 527 °C. Neutron $R_{wp}$: 5.36%.

| **Space Group** | **P-3m1** | | | | | | |
|---|---|---|---|---|---|---|---|
| a (Å) | 5.8955(4) | | alpha (°) | 90 | | | |
| b (Å) | =a | | beta (°) | 90 | | | |
| c (Å) | 16.6179(8) | | gamma (°) | 120 | | | |
| volume (Å$^3$) | 500.21(3) | | | | | | |
| label | Element | Wyckoff | x | y | z | Fractional Occupancy | Uiso |
| Ba1 | Ba | 1 | 0 | 0 | 0 | 1 | 0.0225(10) |
| Ba2 | Ba | 2 | 0.333333 | 0.666667 | 0.8244(6) | 1 | 0.0225(10) |
| Ba3 | Ba | 2 | 0.333333 | 0.666667 | 0.5734(7) | 1 | 0.0225(10) |
| Ba4 | Ba | 2 | 0 | 0 | 0.2824(6) | 1 | 0.0225(10) |
| Nb1 | Nb | 1 | 0 | 0 | 0.5 | 0.76841 | 0.0156(6) |
| Mo1 | Mo | 1 | 0 | 0 | 0.5 | 0.231579 | 0.0156(6) |
| Nb2 | Nb | 2 | 0.333333 | 0.666667 | 0.0951(5) | 0.672368 | 0.0156(6) |
| Mo2 | Mo | 2 | 0.333333 | 0.666667 | 0.0951(5) | 0.202632 | 0.0156(6) |
| V | V | 2 | 0.333333 | 0.666667 | 0.095147 | 0.125 | 0.0156(6) |
| Nb3 | Nb | 2 | 0.333333 | 0.666667 | 0.3506(4) | 0.768421 | 0.0156(6) |
| Mo3 | Mo | 2 | 0.333333 | 0.666667 | 0.3506(4) | 0.231579 | 0.0156(6) |
| O1 | O | 6 | 0.3555(32) | 0.644481 | 0.9854(6) | 0.340(7) | 0.039(8) |
| O2 | O | 6 | 0.1712(10) | 0.828835 | 0.1305(3) | 1.002(7) | 0.0275(5) |
| O3 | O | 6 | 0.1643(10) | 0.835673 | 0.4338(4) | 1 | 0.0275(5) |
| O4 | O | 6 | 0.4944(30) | 0.988898 | 0.29523(28) | 1 | 0.0275(5) |

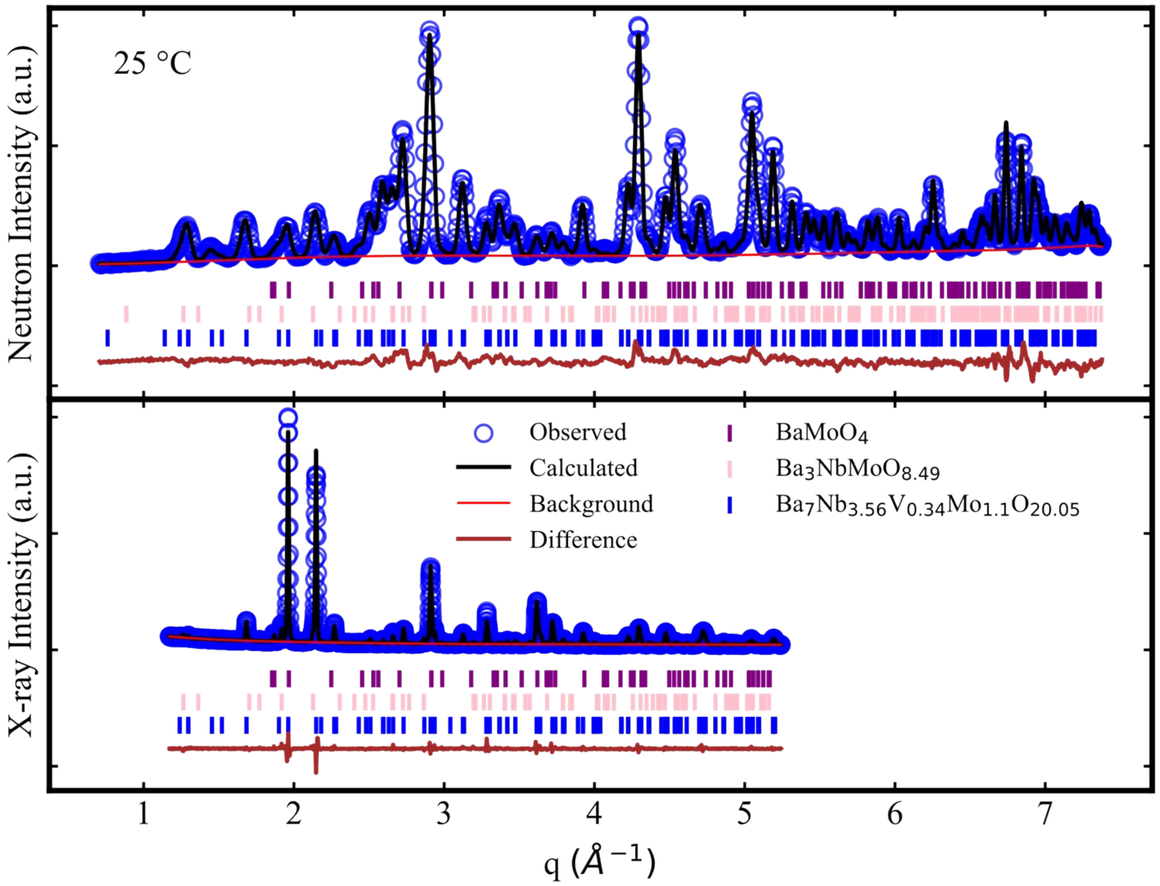


Supporting Figure S9. *A plot of combined Rietveld refinement of both the x-ray and neutron diffraction patterns of* $Ba_7Nb_{3.56}V_{0.34}Mo_{1.1}O_{20.05}$ *at 25 °C (x = 0.34).*

Supporting Table S9. Refined atomic parameters from Rietveld refinement of the combined x-ray and neutron diffraction data of $Ba_7Nb_{3.56}V_{0.34}Mo_{1.1}O_{20.05}$ at 25 °C. X-ray $R_{wp}$: 8.55%, and Neutron $R_{wp}$: 12.80%.

| Space group: | P -3 m 1 | | | | | | |
|---|---|---|---|---|---|---|---|
| a (Å) | 5.85209(18) | | alpha (°) | 90 | | | |
| b (Å) | =a | | beta (°) | 90 | | | |
| c (Å) | 16.5318(4) | | gamma (°) | 120 | | | |
| volume (Å$^3$) | 490.311(15) | | | | | | |
| Label | Element | x | y | z | Fractional Occupancy | Uiso | Wyckoff |
| Ba1 | Ba | 0 | 0 | 0 | 1 | 0.0060(7) | 1 |
| Ba2 | Ba | 0.33333 | 0.66667 | 0.8251(4) | 1 | 0.0060(7) | 2 |
| Ba3 | Ba | 0.33333 | 0.66667 | 0.5737(5) | 1.0066 | 0.0060(7) | 2 |
| Ba4 | Ba | 0 | 0 | 0.2792(3) | 1 | 0.0060(7) | 2 |
| Nb1 | Nb | 0 | 0 | 0.5 | 0.7638 | 0.0025(11) | 1 |
| Mo1 | Mo | 0 | 0 | 0.5 | 0.2362 | 0.0025(11) | 1 |
| Nb2-tet | Nb | 0.33333 | 0.66667 | 0.0969(6) | 0.5718 | 0.0025(11) | 2 |
| Mo2-tet | Mo | 0.33333 | 0.66667 | 0.0969(6) | 0.2573 | 0.0025(11) | 2 |
| V | V | 0.33333 | 0.66667 | 0.0969(6) | 0.1709 | 0.0025(11) | 2 |
| Nb3 | Nb | 0.33333 | 0.66667 | 0.3487(7) | 0.8252 | 0.0025(11) | 2 |
| Mo3 | Mo | 0.33333 | 0.66667 | 0.3487(7) | 0.1748 | 0.0025(11) | 2 |
| O1 | O | 0.6333(28) | 0.3667(28) | 1.0133(13) | 0.341667 | 0.010(9) | 6 |
| O2 | O | 0.1712(18) | 0.8288(18) | 0.1332(7) | 1 | 0.0099(9) | 6 |
| O3 | O | 0.1665(21) | 0.8335(21) | 0.4333(8) | 1 | 0.0099(9) | 6 |
| O4 | O | 0.494(6) | 0.989(12) | 0.2945(6) | 1 | 0.0099(9) | 6 |

| Impurity Phases | | | |
|---|---|---|---|
| $BaMoO_4$ | | Space group: I41/a | |
| a=b (Å) | 5.584(2) | alpha=beta= gamma (°) | 90 |
| c (Å) | 12.827(5) | mass fraction | 0.0137(6) |
| volume (Å$^3$) | | 399.900(3) | |
| $Ba_3V_xNb_{2-x}O8$ | | Space group: R-3m1 | |
| a=b (Å) | 5.906(1) | alpha=beta (°) | 90 |
| c (Å) | 21.321(4) | gamma (°) | 120 |
| volume (Å$^3$) | 644.200(1) | | |
| mass fraction | | 0.0355(9) | |

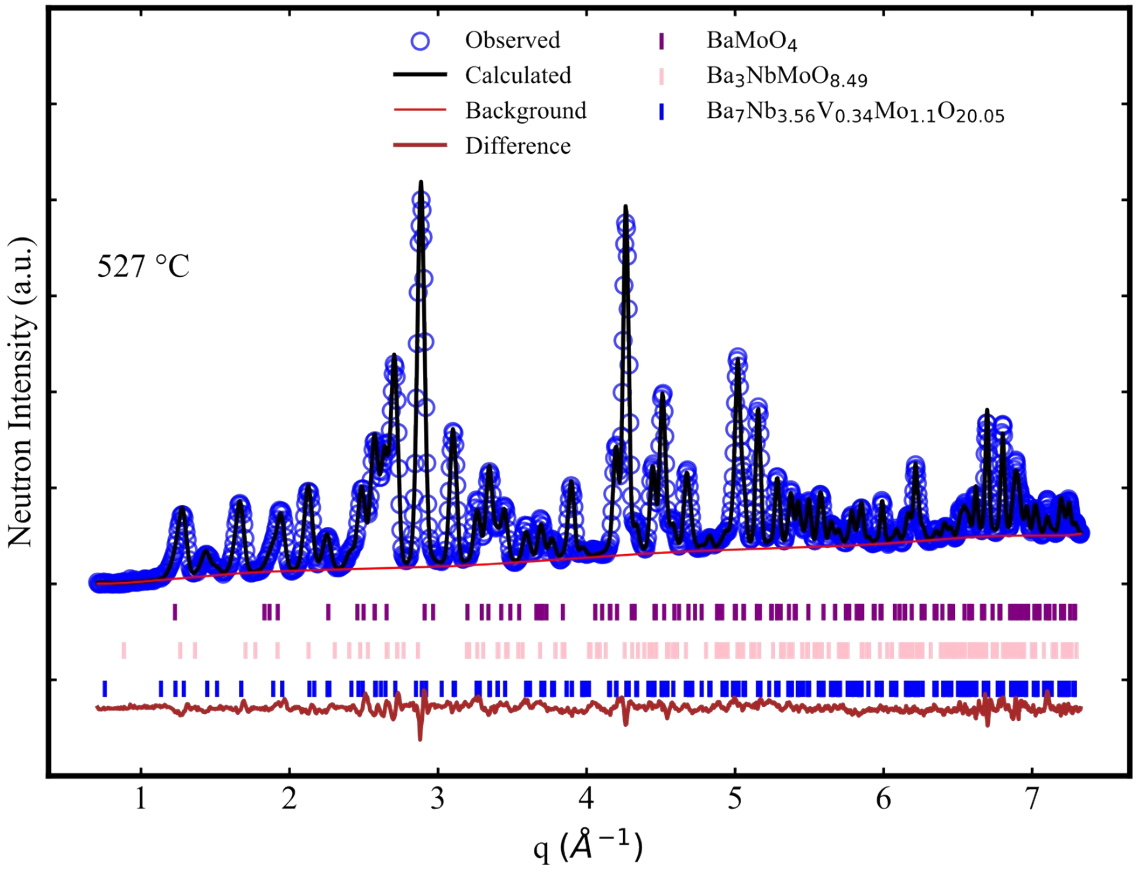


Supporting Figure S10. *A plot of Rietveld refinement of neutron diffraction patterns of* $Ba_7Nb_{3.56}V_{0.34}Mo_{1.1}O_{20.05}$

Supporting Table S10. Refined atomic parameters from Rietveld refinement of neutron diffraction data of $Ba_7Nb_{3.56}V_{0.34}Mo_{1.1}O_{20.05}$ at 527 °C. Neutron $R_{wp}$: 6.43%.

| **Space Group** | **P-3m1** | | | | | | |
|---|---|---|---|---|---|---|---|
| a (Å) | 5.8898(4) | | alpha (°) | 90 | | | |
| b (Å) | 5.8898(4) | | beta (°) | 90 | | | |
| c (Å) | 16.6196(9) | | gamma (°) | 120 | | | |
| volume (Å$^3$) | 499.29(4) | | | | | | |
| Label | Element | Wyckoff | x | y | z | Fractional Occupancy | Uiso |
| Ba1 | Ba | 1 | 0 | 0 | 0 | 1 | 0.0238(9) |
| Ba2 | Ba | 2 | 0.33333 | 0.66667 | 0.8233(6) | 1 | 0.0238(9) |
| Ba3 | Ba | 2 | 0.33333 | 0.66667 | 0.5731(6) | 1 | 0.0238(9) |
| Ba4 | Ba | 2 | 0 | 0 | 0.2817(6) | 1 | 0.0238(9) |
| Nb1 | Nb | 1 | 0 | 0 | 0.5 | 0.763847 | 0.0143(5) |
| Mo1 | Mo | 1 | 0 | 0 | 0.5 | 0.236153 | 0.0143(5) |
| Nb2 | Nb | 2 | 0.33333 | 0.66667 | 0.0944(5) | 0.588(7) | 0.0143(5) |
| Mo2 | Mo | 2 | 0.33333 | 0.66667 | 0.0944(5) | 0.182(7) | 0.0143(5) |
| V | V | 2 | 0.333333 | 0.666667 | 0.094424 | 0.171 | 0.0143(5) |
| Nb3 | Nb | 2 | 0.33333 | 0.66667 | 0.3489(3) | 0.763847 | 0.0143(5) |
| Mo3 | Mo | 2 | 0.33333 | 0.66667 | 0.3489(3) | 0.236153 | 0.0143(5) |
| Nb4 | Nb | 2 | 0.33333 | 0.66667 | 0.208(6) | 0.045(7) | 0.0143(5) |
| Mo4 | Mo | 2 | 0.33333 | 0.66667 | 0.208(6) | 0.014(7) | 0.0143(5) |
| O1 | O | 6 | 0.3054(22) | 0.694568 | 0.9887(6) | 0.342 | 0.040(6) |
| O2 | O | 6 | 0.1705(9) | 0.829493 | 0.13212(29) | 1 | 0.0265(4) |
| O3 | O | 6 | 0.1639(8) | 0.836064 | 0.4325(4) | 1 | 0.0265(4) |
| O4 | O | 6 | 0.4925(28) | 0.984981 | 0.29492(26) | 1 | 0.0265(4) |

Supporting Table S11. The value of simulated CQ and CSA tensor parameters.

| *Sample* | *Spinning Frequency (kHz)* | *CQ (MHz)* | $\eta_Q$ | $\delta$*(ppm)* | $\eta$ | $\varphi^a$ (°) | $\theta^a$ (°) | $\psi^a$ (°) | *Comment* |
|---|---|---|---|---|---|---|---|---|---|
| $V_2O_5$ | 10 | 1.0 ± 0.1 | 0.7 ± 0.1 | -895 ± 5 | 0.2 ± 0.1 | 270 | - | - | Combined CQ and CSA fit |
| $Ba_7Nb_{3.8}V_{0.1}MoO_{20.05}$ | 10 | 0.9 ± 0.1 | 0.8 ± 0.1 | <135 ± 5[b] | 1.0 ± 0.1 | - | - | - | Individual CQ fit |
| $Ba_7Nb_{3.7}V_{0.2}MoO_{20.05}$ | 10 | 0.8 ± 0.1 | 0.9 ± 0.1 | <132 ± 5[b] | 1.0 ± 0.1 | - | - | - | Individual CQ fit |
| $Ba_7Nb_{3.6}V_{0.3}MoO_{20.05}$ | 10 | 0.8± 0.1 | 0.9 ± 0.1 | <125 ± 5[b] | 0.9 ± 0.1 | - | - | - | Individual CQ fit |
| $Ba_7Nb_{3.5}V_{0.4}MoO_{20.05}$ | 10 | 0.8 ± 0.1 | 0.9 ± 0.1 | <128 ± 5[b] | 1.0 ± 0.1 | - | - | - | Individual CQ fit |
| $Ba_7V_{3.9}MoO_{20.05}$ | 33 | 0.9 ± 0.1 | 0.6 ± 0.1 | <454 ± 5 [bc] | 0.9 ± 0.1 | - | - | - | Individual CQ fit |

a: Euler angles not determined in the fit. (angles set to zero, assuming the CSA and EFG tensor are aligned).

b: highest threshold for the reduced anisotropy of the CSA tensor. Estimated from a simulation taking only the CSA into account (i.e. assuming CSA is dominant, and no contribution from $C_Q$ present), and aiming to reproduce the intensity ratio between centreband and the first set of spinning sidebands flanking it. Any larger CSA values would increase the intensity of spinning sidebands, and hence lead to a poorer fit.

c: given the higher spinning frequency (33 kHz instead of 10 kHz) and the correspondingly larger distance between first set of spinning sidebands and the centreband will result in a higher threshold for the lowest CSA tensor able to describe their intensity ratio.

Supporting Table S12. Equilibrium oxygen sites along with their respective energies and occupancies, and the oxygen sites from combined XRD and neutron diffraction refinement of $Ba_7N_{b3.9}Mo_{1.1}O_{20.05}$

| BVSE | | | | | | | | |
|---|---|---|---|---|---|---|---|---|
| Site | Occupancy | Multiplicity | X | Y | Z | Bond Valence Sum | Coordination Number | Energy |
| O5’ | 0.471 | 6.000 | 0.486 | 0.514 | 0.018 | -1.521 | 4.000 | 0.710 |
| O2 | 1.000 | 12 | 0.166 | 0.333 | 0.137 | -1.767 | 5.000 | 0.758 |
| O3 | 1.000 | 12 | 0.166 | 0.333 | 0.429 | -1.937 | 6.000 | 0.000 |
| O4 | 1.000 | 6.000 | 0.486 | 0.514 | 0.292 | -2.041 | 5.000 | 0.596 |
| s1 | 0 | 3 | 0.5 | 0 | 0 | - | - | - |
| s2 | 0 | 6 | 0.736 | 0.264 | 0.012 | - | - | - |
| Rietveld Refinement | | | | | | | | |
| Site | Occupancy | Multiplicity | X | Y | Z | Bond Valence Sum | Coordination Number | |
| O1 | 0.258 | 6 | 0.333333 | 0.666667 | 0.1903(2) | -1.984 | 1.913 | |
| O2 | 1.000 | 6 | 0.1691(1) | 0.830915 | 0.1319(3) | -1.863 | 5.000 | |
| O3 | 1.000 | 6 | 0.1638(9) | 0.836237 | 0.4320 | -1.955 | 6.000 | |
| O4 | 1.000 | 6 | 0.493(3) | 0.986787 | 0.29479(2) | -2.000 | 5.091 | |
| O5 | 0.167 | 3 | 0.500 | 0.000 | 0.000 | -1.356 | 5.830 | |

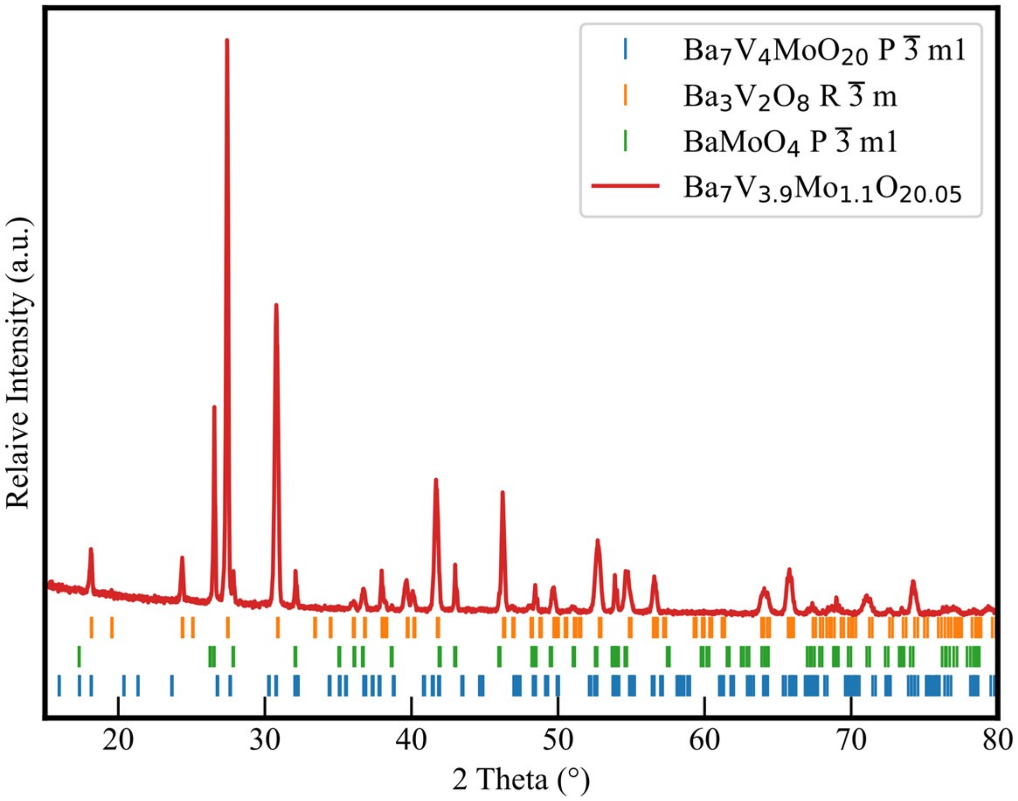


Supporting Figure S11. *A plot of the XRD pattern of* $Ba_7V_{3.9}Mo_{1.1}O_{20.05}$.

## $^{51}V$ solid-state NMR

$^{51}V$ MAS NMR spectra are a result of the convolution of structure-related nuclear interactions such as the quadrupolar coupling [4] and chemical shift anisotropy (CSA) [5], which determine the position and shape of signals in the spectra, and under MAS, the extent of spinning sidebands envelope [6–10]. The quantification of these two interactions provides information about the local structure of vanadium in inorganic compounds [7,8].

Due to the modest magnitude of the electric quadrupolar moment for $^{51}V$, quite often, $^{51}V$ MAS NMR spectra are dominated by CSA tensor parameters, namely, chemical shift anisotropy (CSA), with its reduced anisotropy (δ) and asymmetry ($\eta_\sigma$) [11] (in the Haeberlen convention) [12]. Given the prominent role of CSA tensor parameters in $^{51}V$ NMR spectra, the evaluation of these parameters plays a key role in the identification of the coordination environment for vanadium [11]. NMR parameters of $^{51}V$ MAS spectra have been empirically correlated to the coordination environment of vanadium in several oxide systems [7,11].

The reported correlation allows the identification of three types of tetrahedral environment for Vanadium: distorted isolated tetrahedron (type $Q^0$), strongly distorted tetrahedral environment associated with adjacent tetrahedral by one (type $Q^1$) or two bridge (type $Q^2$) oxygen atoms [7]. Vanadium in the pyramidal coordination environment are distinguished by correlating both the so-called effective value of CSA and the $C_Q$ tensor parameters in a case where both $\delta > 200$ ppm and $\eta_\sigma < 0.6$ [7]. The values of both $\delta$ and $\eta_\sigma$ have been computed from the fitting of experimental data for several compounds, and it was observed that similar types of $VO_x$ species have similar CSA parameters [7,11].

$^{51}V$ is an I=7/2 nucleus, and with $C_Q$ values usually reported in the order of a few MHz [8,9], effects from quadrupolar interaction are expected only to first-order perturbation. Hence, under MAS, a centre band consisting of the central transition (m=1/2→-1/2) is expected, which is flanked by a series of spinning sidebands corresponding to the satellite transitions. Given the similar magnitude of the CSA, in frequency units, when compared to the quadrupolar frequency, two scenarios can be expected. In case the CSA has a negligible contribution to the spectrum (*i.e.* $\delta$< 400 ppm), a centre band with high intensity is flanked by a series of low-intensity spinning sidebands of the satellites. However, should the CSA have a similar magnitude as the first-order quadrupolar interaction (*i.e.* $\delta$ > 400 ppm), the central transition is also broadened non-homogeneously by this interaction, and hence, the intensity from the central transition is smeared out into the spinning sidebands, with no longer a distinct intensity for the isotropic centre-band. In fact, depending on the symmetry of the CSA tensor, spinning sidebands other than the isotropic centre band may exhibit the highest intensity in the spectrum, and measurements at different MAS frequencies are necessary to unambiguously identify the latter.

A typical spectrum of $^{51}V$ in an octahedral environment can be observed for $V_2O_5$, a compound often employed as a secondary reference for the $^{51}V$ chemical shift scale. The following values of $C_Q$ and CSA tensor parameters have been reported for $^{51}V$ MAS spectrum of $V_2O_5$: $C_Q$ = 0.987 MHz, $\eta_Q$ = 0, $\delta$ = 645 ppm, and $\eta_\sigma$ = 0.11 [13]. These values indicate a similar contribution of quadrupolar interaction and CSA to the spectrum, in particular for a $B_0$ field of 9.4 T. The experimental and simulated $^{51}V$ MAS spectra of $V_2O_5$ are presented in Fig. S12. The experimental spectrum has an isotropic line (denoted by an arrow) flanked by spinning sideband patterns. This pattern of spinning sidebands cannot be reproduced by only taking the quadrupolar interaction into account. A

satisfactory simulation is achieved by a combined fit of both $C_Q$ and CSA tensor parameters, which matches well with literature values (Fig. S12).

It is to be noted that distorted octahedral or five-coordinate environments of vanadium in pyro-and metavanadates, as well as $V_2O_5$, have been reported to exhibit large value of $\delta$ ranging from |295| to |640| ppm [14–16]. These correlations are consistent with the trend reported by Lapina *et al.* [7]; thus, supporting the use of $^{51}V$ MAS NMR spectra parameters in the determination of the local structure of vanadium in vanadium compounds.

In the next step, the interpretation obtained from $^{51}V$ MAS NMR spectrum parameters of $V_2O_5$ is applied to the $^{51}V$ MAS NMR spectrum acquired for $Ba_7Nb_{3.8}V_{0.1}Mo_{1.1}O_{20.05}$ (Fig. S13). The isotropic line is marked with an arrow and this line is flanked by symmetric spinning sidebands of much lower intensity.

In a further attempt, it is readily apparent that the parameters that simulated the experimental spectrum properly is the $C_Q$; thus, signifying that $C_Q$ is the most relevant perturbation for vanadium nucleus in $Ba_7Nb_{3.8}V_{0.1}Mo_{1.1}O_{20.05}$. Moreover, the fact that CSA was not needed to simulate these spectra indicates a small magnitude for this interaction, in the order of 135.50 ppm or smaller.

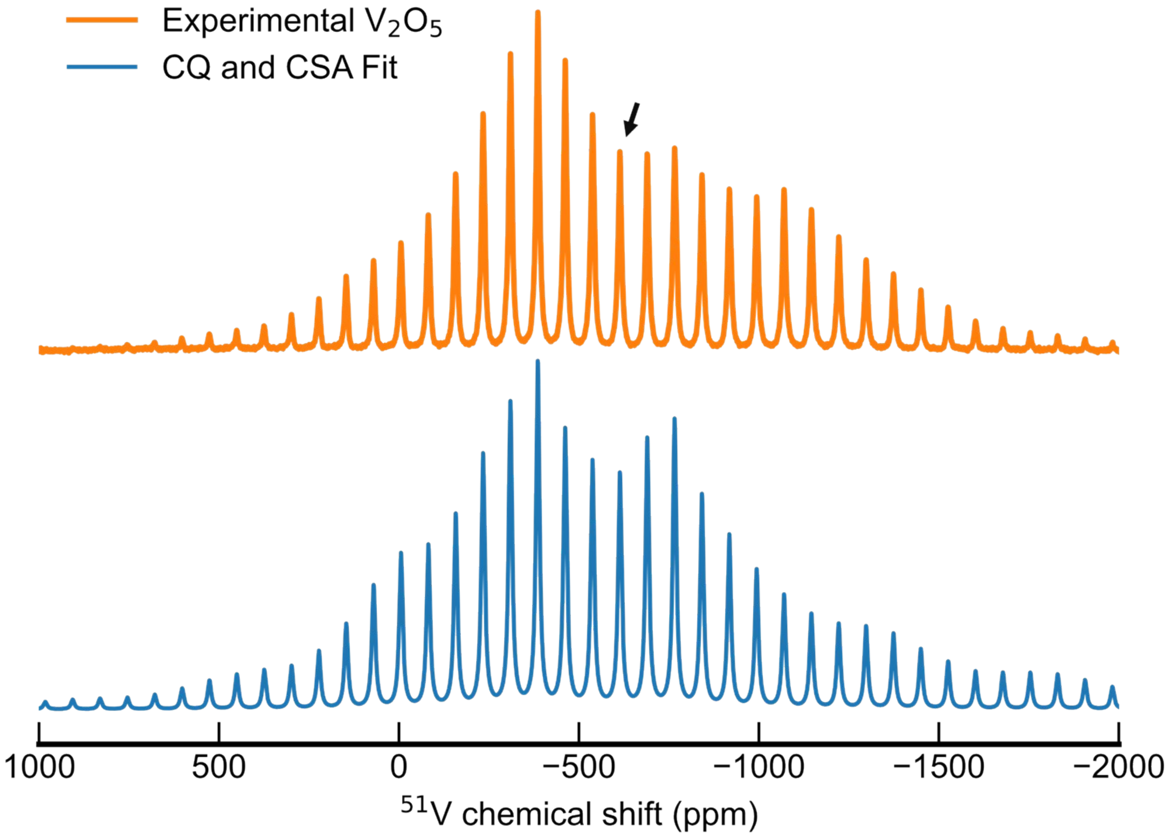


*Supporting Fig. S12. $^{51}V$ MAS NMR spectra spun at 10 kHz of $V_2O_5$ spectrum fitted with both $C_Q$ parameters and CSA parameters. The arrow indicates the isotropic chemical shift of $^{51}V$ in $V_2O_5$.*

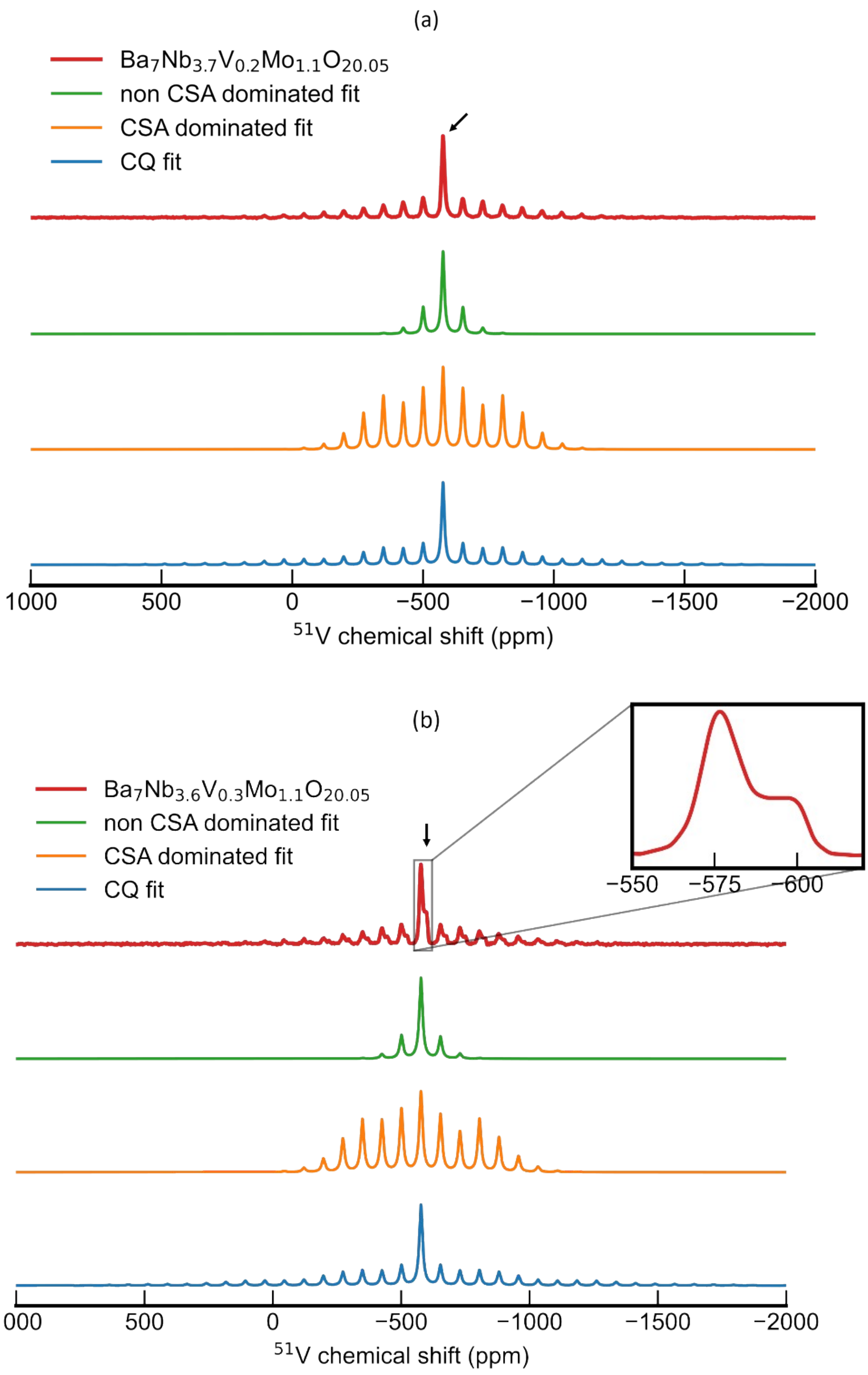
(a)
Ba7Nb3.7V0.2Mo1.1O20.05
non CSA dominated fit
CSA dominated fit
CQ fit
1000
500
0
−500
−1000
−1500
−2000
51V chemical shift (ppm)
(b)
Ba7Nb3.6V0.3Mo1.1O20.05
non CSA dominated fit
CSA dominated fit
CQ fit
−550
−575
−600
1000
500
0
−500
−1000
−1500
−2000
51V chemical shift (ppm)

(c)

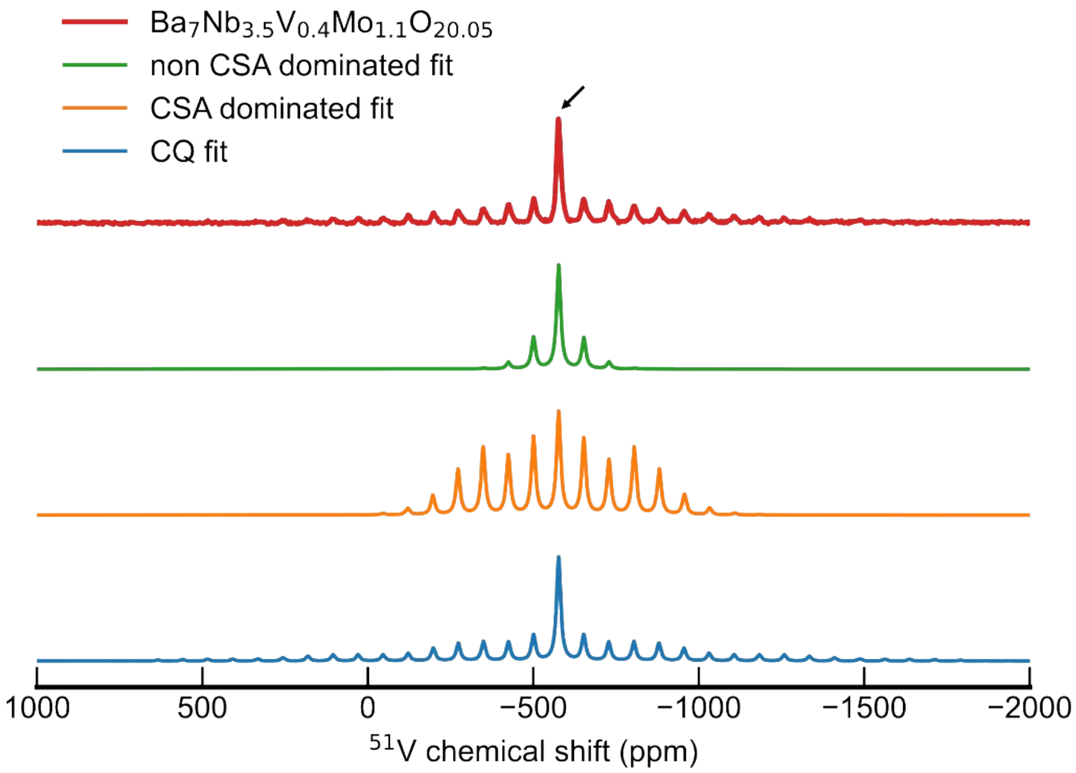


Supporting Figure S13. *$^{51}V$ NMR spectra spun at 10 kHz of $Ba_7Nb_{3.9-x}V_xMo_{1.1}O_{20.05}$ (0.1≤x≤0.4) spectra fitted with only CSA parameters, and only CQ parameters. The isotropic chemical shift is indicated with a black arrow.*

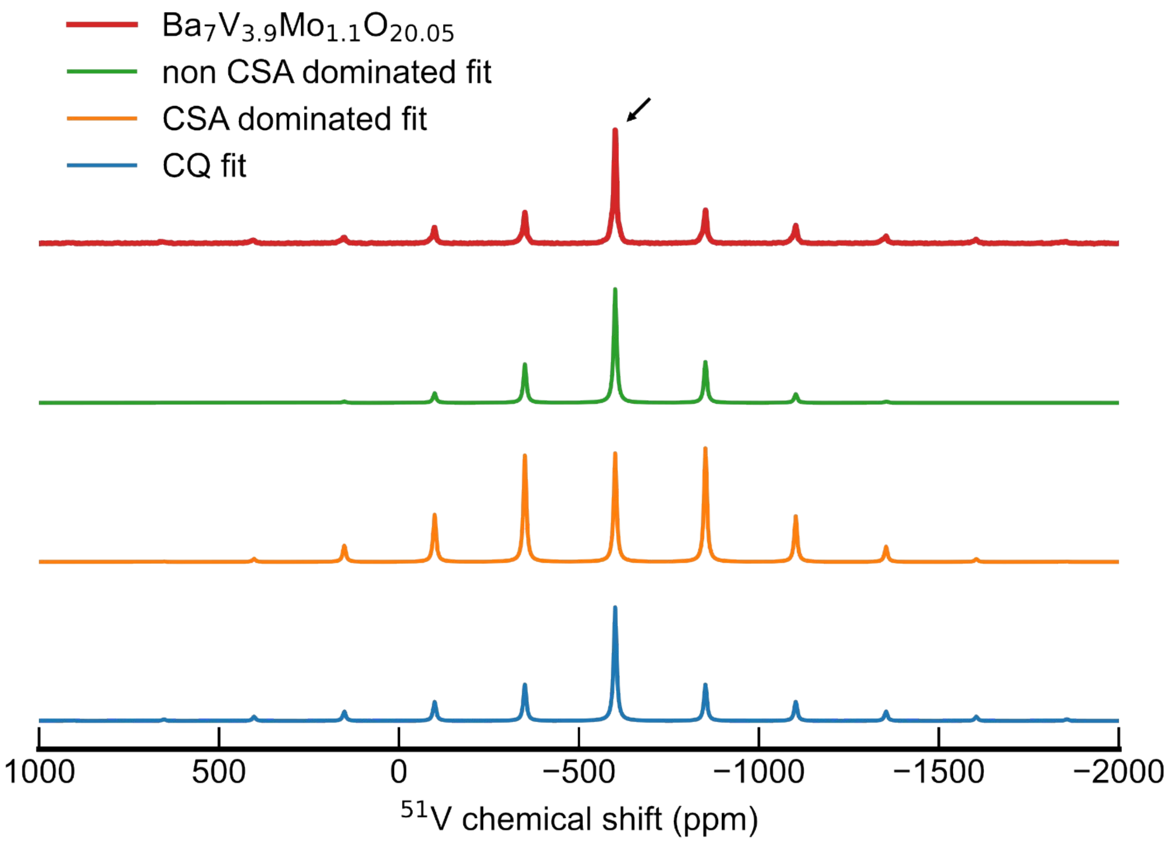


Supporting Figure S14. *$^{51}V$ NMR spectra spun at 33 kHz of $Ba_7V_{3.9}Mo_{1.1}O_{20.05}$ spectra fitted with only CSA parameters, and only CQ parameters. The isotropic chemical shift is indicated with a black arrow. The $Ba_7V_{3.9}Mo_{1.1}O_{20.05}$ is a nominal composition that contains predominantly the $Ba_3V_2O_8$ phase.*

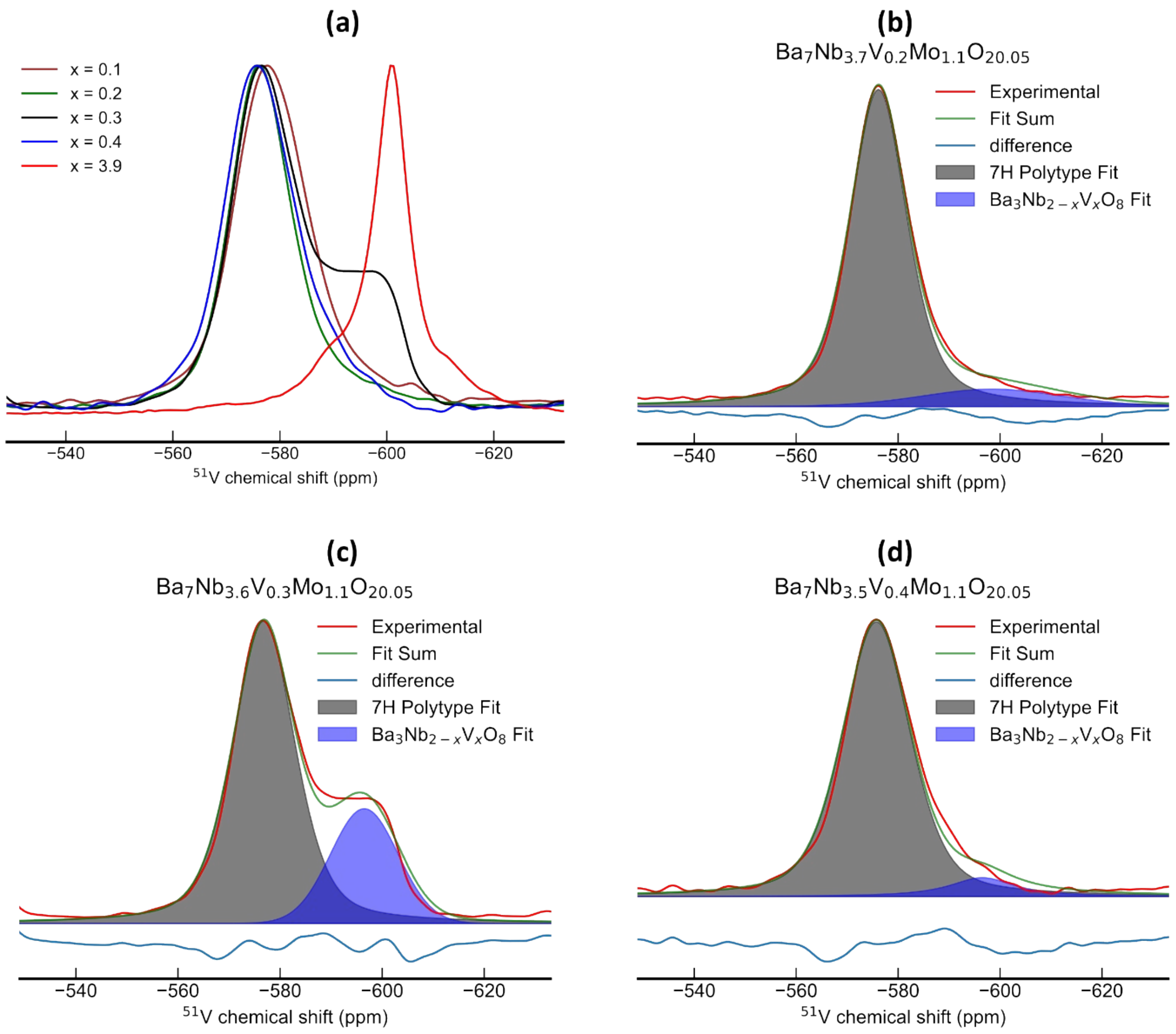


Supporting Fig. S15. *(a) A zoomed plot on the isotropic lines of the* $^{51}V$ *MAS NMR of the synthesized* $Ba_7Nb_{3.9-x}V_xMo_{1.1}O_{20.05}$ *series, (b-d) integrated spectra of synthesised* $Ba_7Nb_{3.9-x}V_xMo_{1.1}O_{20.05}$ *(0.2≤x≤0.4) series.*

From figure Fig. S15a, it was noted that $Ba_3Nb_{2-x}V_xO_{8.5}$ side phase is present in the synthesised $Ba_7Nb_{3.9-x}V_xMo_{1.1}O_{20.05}$ series from the range 0.2≤x≤0.4. The spectra in this range of 0.2≤x≤0.4 were integrated within the chemical shift region of -620 to -560 ppm for the quantification of the percentage of both the 7H-polytpe and vanadium-rich $Ba_3Nb_{2-x}V_xO_{8.5}$ side phase. The intensity of the $Ba_3Nb_{2-x}V_xO_{8.5}$ resonance at around -577ppm increases upon V-doping; however, there is a significant drop in this intensity between 0.3 and 0.4 value of x. Values of apparent chemical shift are reported. Quadrupolar induced shift lower than 0.1 ppm are expected for the lines given the $C_Q$ in the order of 1 MHz and the B0 field of 11.4 T employed in the experiments.

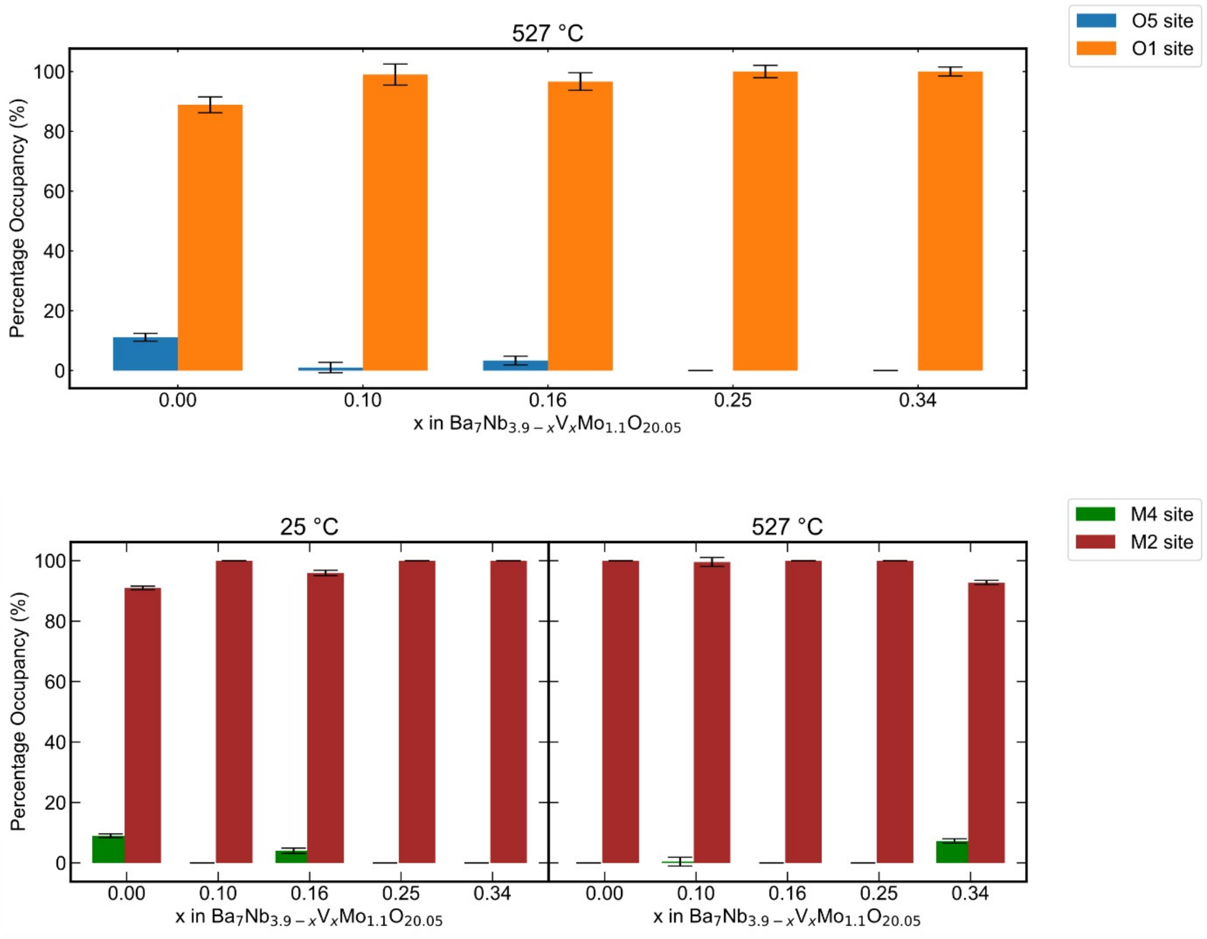


Supporting Figure S16. *Bar plots depicting the percentage occupancy of oxide-ion disorder at 527°C, and M2 and M4 sites in* $Ba_7Nb_{3.9-x}V_xMo_{1.1}O_{20.05}$ *series at 25 °C and 527 °C, respectively.*

## BVSE

In a bid to understand the differences in the energy landscape of each member of the $Ba_7Nb_{3.9-x}V_xMo_{1.1}O_{20.05}$ series, the *softBV* calculation is extended to the structural model of $Ba_7Nb_{3.9}Mo_{1.1}O_{20.05}$ reported by Yashima *et al.* [17]. With this calculation, $Nb^{5+}$ is subsequently replaced by $V^{5+}$ to generate the $Ba_7Nb_{3.9-x}V_xMo_{1.1}O_{20.05}$ ($0 \leq x \leq 0.8$) series. The nominal composition of vanadium is then ordered at M1, M2, and M3 average crystallographic sites, respectively, and the oxygen energy landscape was subsequently calculated. Fig. S17a illustrates the BVSE landscape for the $Ba_7Nb_{3.9-x}V_xMo_{1.1}O_{20.05}$ ($0 \leq x \leq 0.8$) series generated from Yashima *et al.* [17] model with the nominal composition of vanadium ordered at M2 site. Fig. S17b depicts the evolution of the relative energy of the s2 saddle point across the $Ba_7Nb_{3.9-x}V_xMo_{1.1}O_{20.05}$ ($0 \leq x \leq 0.8$) series where the nominal composition of V is ordered at M1, M2 or M3 site.

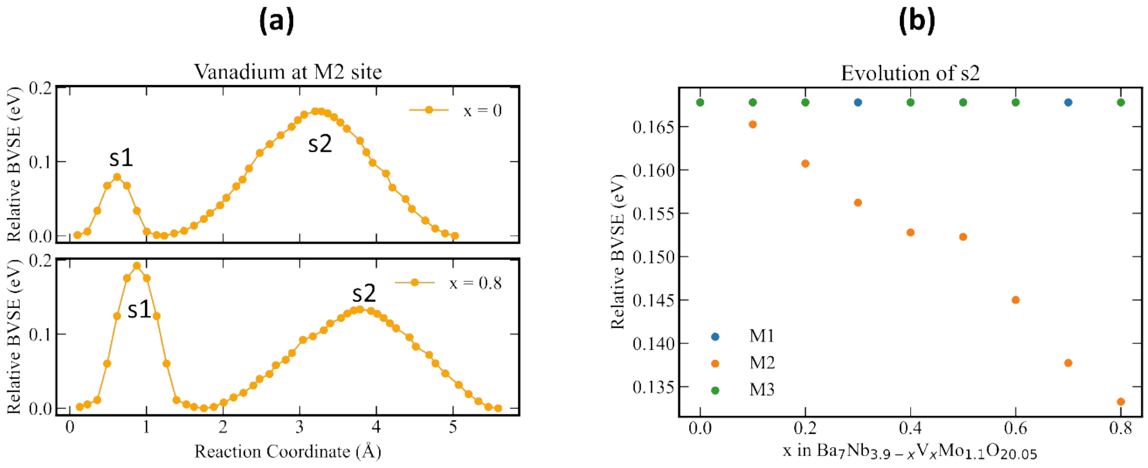


Supporting Figure S17. *(a) The BVSE landscape for the $Ba_7Nb_{3.9-x}V_xMo_{1.1}O_{20.05}$ (x = 0 & 0.8) series generated from Yashima et al.* [17] *model with the nominal composition of vanadium ordered at M2 site, and (b) the evolution of the relative energy of the s2 saddle point across the $Ba_7Nb_{3.9-x}V_xMo_{1.1}O_{20.05}$ ($0 \leq x \leq 0.8$) series when the nominal composition of V is ordered at M1, M2 or M3 site.*

From *softBV* calculation of oxide-ion conduction in the average structure of $Ba_7Nb_{3.9-x}V_xMo_{1.1}O_{20.05}$ ($0 \leq x \leq 0.8$) series based on the Yashima *et al.* [17] model, it was noted that ordering of the nominal composition of vanadium on either M1 or M3 site and then subsequently increasing vanadium content have no effect on the BVSE landscape (Fig. S17b). The relative bond-valence site energy of s2 saddle points of both M1 and M3 site ordering of nominal composition of vanadium did not change upon increasing vanadium content (Fig. S17b). However, ordering the nominal composition of vanadium at average crystallographic M2 site, and subsequently increasing its content, has a pronounced effect on the relative BVSE of the s2 saddle points (Fig. S17a). The relative BVSE of the s2 saddle point, for the situation where the nominal composition of vanadium is placed at M2 site, decreases linearly with increasing vanadium content (Fig. S17b). Thus, it can be rationalised that the key factor impacting oxide-ion conduction in the $Ba_7Nb_{3.9-x}V_xMo_{1.1}O_{20.05}$ is the presence of the nominal composition of vanadium at the average M2 crystallographic site.

## Notes on sample stoichiometry and cation content

$MoO_3$ is a volatile oxide, with non-negligible vapour pressure above 600 $^{o}C$. However, it reacts readily with $BaCO_3$ above 500 $^{o}C$ forming $BaMoO_4$ [18]. This point was also observed in X-ray diffraction patterns of the first calcination steps, which show $BaMoO_4$ as a side phase and potential reaction intermediate. Based on the above, we did not expect considerable loss for neither Mo nor Ba during this reaction, in spite of reported volatility of both BaO and $MoO_3$ at the calcination temperature used. This expectation is supported by the Rietveld refinement from diffraction data, in particular XRD, which does not indicate any major cation deficiencies.

In spite of that, we were interested whether further chemical analysis would be able to provide additional insight about the stoichiometric coefficients for the oxides synthesized. Hence, the cation content of two batches of the undoped composition was measured with ICP-MS.

Notably, ICP-MS of oxides based on Ba, Mo and Nb is far from trivial, and very prone to inaccuracies both stemming from sample dissolution as well as cross-species interferences. In detail, one may expect interferences of $^{93}Nb$ with $^{92}Mo^{1}H^{+}$ to arise. Furthermore, Ba in presence of Mo may form insoluble molybdates after dissolution/dilution procedures. In addition to that, Ba may also interfere with $F^{-}$ from HF, which was required to fully dissolve these oxides. At last, due to Nb oxides formed during dissolution can be very resistant to acids, including HF, and would require non-standard dissolution procedures, such as fusion with $K_2S_2O_7$, which are not widespread available.

In detail, dissolution of both samples was attempted either with aqua regia (AR), or a mixture of aqua regia and hydrofluoric acid (AR + 0.4HF). Results from these measurements are given in Table S13a and b. Approximately 20 mg sample was digested in 1.5 ml 65% $HNO_3$ and 4.5 ml 30% HCl and 0.0 ml 40% HF resp 0.4 ml 40% HF, using the microwave. The digestion time in the microwave was 60 min. at max power. After destruction, the sample was diluted to 50 ml with Milli-Q water. The samples were diluted in 1%$HNO_3$. The samples were analysed with ICP-MS Perkin Elmer Nexion 2000 at the TU Delft Reactor Institute.

Table S13a: Raw data stemming from ICP-MS measurements for samples with x = 0.

| **AN2024.04.004** | **weight [mg]** | **Nb [wt%]** | **Mo [wt%]** | **Ba [wt%]** | destruction |
|---|---|---|---|---|---|
| AO13 | 23,93 | 3,7% | 4,4% | 47,5% | AR |
| AO18 | 28,20 | 12,4% | 5,1% | 44,7% | AR |
| AO13 | 23,66 | 23,4% | 6,0% | 57,7% | AR+0,4HF |
| AO18 | 26,90 | 21,9% | 5,8% | 56,3% | AR+0,4HF |

Table S13b: Analysis of ICP-MS data for samples x=0.

| | Ba | Nb | Mo | O |
|---|---|---|---|---|
| stoichiometric coefficients | 7,0 | 3,9 | 1,1 | 20,1 |
| atomic weight | 137,3 | 92,9 | 96,0 | 16,0 |
| Stoich*MW | 961,3 | 362,3 | 105,5 | 320,8 |
| expected weight fraction | 54,9 | 20,7 | 6,0 | 18,3 |
| ICP - Aqua Regia (AR) | | | | |
| A013_AR - weight fraction | 47,5 | 3,7 | 4,4 | 44,4 |
| A013_AR_stoichiometric coefficients | 6,1 | 0,7 | 0,8 | 48,6 |
| A018_AR - weight fraction | 44,7 | 12,4 | 5,1 | 37,8 |
| A018_AR_stoichiometric coefficients | 5,7 | 2,3 | 0,9 | 41,3 |
| ICP - Aqua Regia + HF | | | | |
| A013_AR_0p4HF - weight fraction | 57,7 | 23,4 | 6,0 | 12,9 |
| A013_AR_0p4HF_stoichiometric coefficients | 7,4 | 4,4 | 1,1 | 14,1 |
| A018_AR_0p4HF | 56,3 | 21,9 | 5,8 | 16,0 |
| A018_AR_0p4HF_stoichiometric coefficients | 7,2 | 4,1 | 1,1 | 17,5 |

From the ICP-MS results, the digestion with a cocktail of Aqua-regia and HF seems to work better. And from the calculated stoichiometry of each element in both batches, it is evident that there seems not to be a Mo-deficiency.

## $^{93}$Nb MAS NMR Spectroscopy

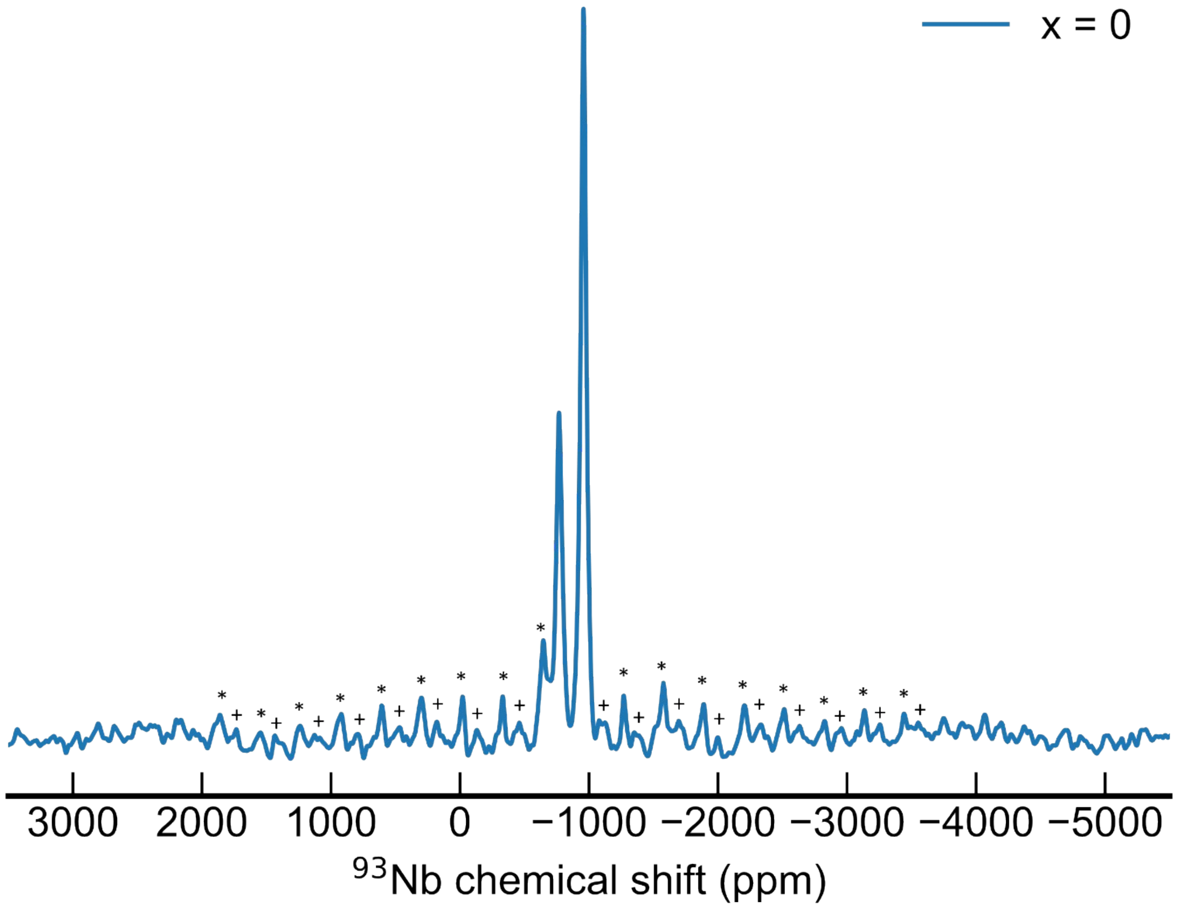


Supporting Figure S18 $^{93}$Nb: full spectrum showing spinning sidebands from satellites, and how 38 kHz MAS at the field of 11.4 T is enough to resolve both central transitions from their respective spinning sidebands.

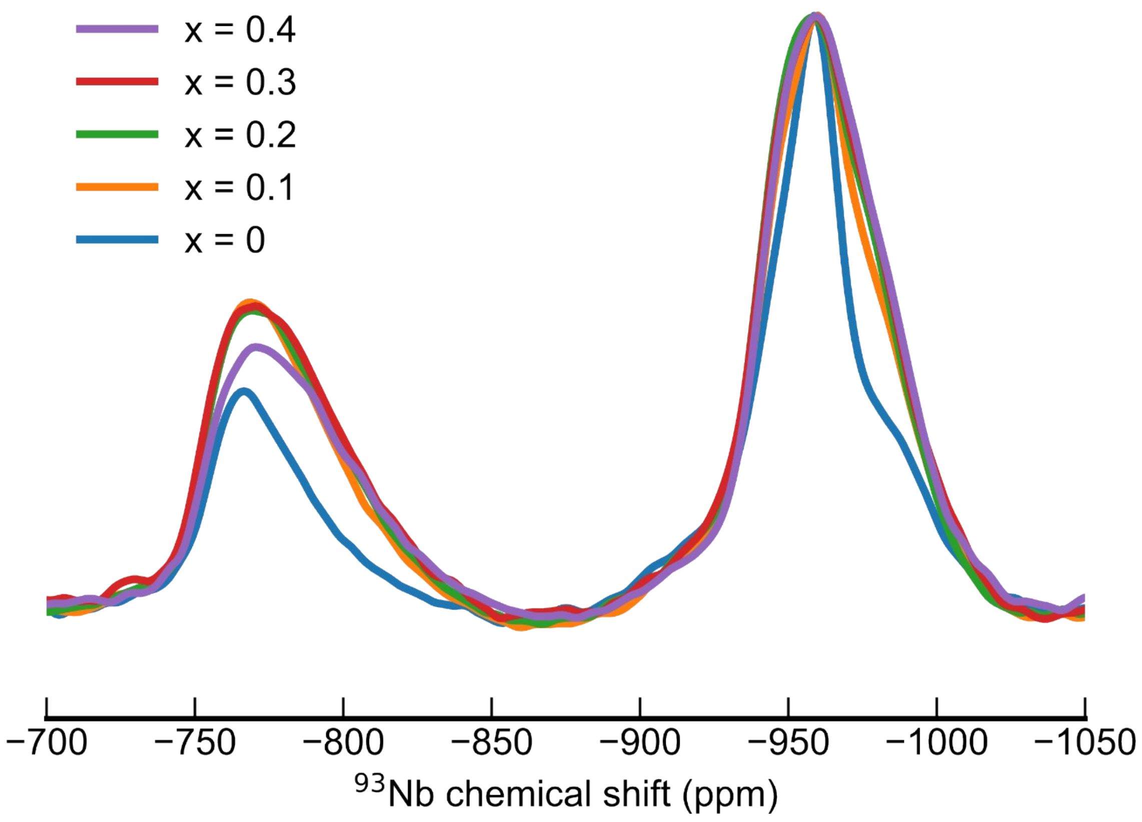


Supporting Figure S19 $^{93}$Nb NMR: Overlay of central transition region for composition x=0 up to x=0.4

## Calibration Temperature NPD measurements

For the high-temperature neutron diffraction experiments, we employed , the hot stage developed for the PEARL neutron powder diffractometer at the TU Delft reactor institute. The sample heater consists of a copper resistive heating element and vanadium foil wrapped around the sample can. Sample temperature was determined indirectly via a thermocouple placed within the copper block, which is connected to the heating element (Fig. S20a). As for its calibration, the temperature difference between the copper block and sample was determined by measurement with a second thermocouple, inserted within a reference sample of $Al_2O_3$. This procedure resulted in the calibration curve depicted in Fig. S20b. For the high-temperature measurements on hexagonal perovskite samples, temperature of copper block thermocouple was set to 830 K, which taking into account the calibration curve, should result in a samples temperature of 800±5 $^{o}$C.

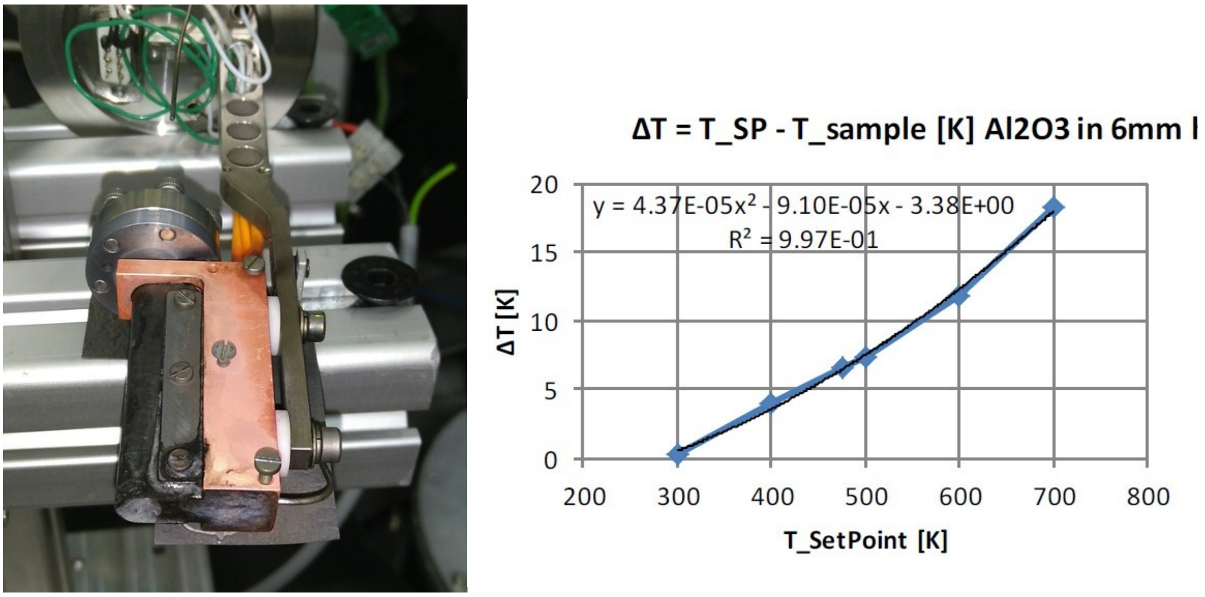


Supporting Figure S20: (a) Block of copper metal along with Vanadium foil wrapping, used as heating set up for the Vanadium can and its content during the HT (800 K) NPD experiment at PEARL – TU Delft Reactor Institute, the Netherlands. (b) Calibration curve between copper block thermocouple and sample temperature.

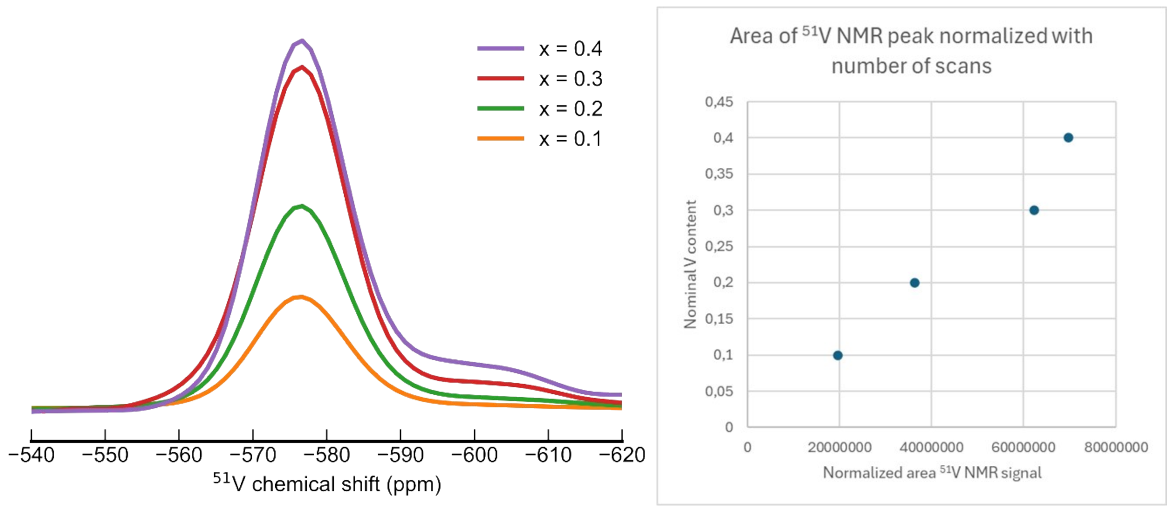


Supporting Figure 21: $^{51}V$ MAS NMR spectra (only CT shown) normalized with respect to number of scans. A clear trend of increasing $^{51}V$ signal area against nominal V content can be observed.

Figure S21 shows $^{51}V$ MAS NMR spectra (only CT shown) normalized with respect to number of scans. A different number of scans are required to attain a similar signal-to-noise ratio in the 51V NMR spectra of samples with varying vanadium content. Sample holders (rotors) have been filled to the same height, hence sample weight for this series of measurements in comparable. An increase in peak height (and area) as a function of (nominal) vanadium content can be observed. The same trend is observed in a plot of signal area against nominal V content. However, one reservation to this approach is the variability in NMR rotor mass loading, which was not tracked and may contribute to non-negligible uncertainty in the NMR signal area.

## Bibliography


[1] A. Denton, N. Ashcroft, Vegard's law, Phys. Rev. A 43 (1991) 3161–3164. https://doi.org/10.1103/PhysRevA.43.3161.

[2] S. Kemmler-Sack, U. Treiber, Über hexagonale Perowskite mit Kationenfehlstellen. XXVIII. Die Struktur der rhomboedrischen 9 L-Stapelvarianten Ba3WNb□O9−x/2□x/2, Zeitschrift für anorganische und allgemeine Chemie 478 (1981) 198–204. https://doi.org/10.1002/zaac.19814780719.

[3] G. Liu, J.E. Greedan, Syntheses, Structures, and Characterization of 5-Layer BaVO3-x (x = 0.2, 0.1, 0.0), Journal of Solid State Chemistry 110 (1994) 274–289. https://doi.org/10.1006/jssc.1994.1170.

[4] D. Freude, J. Haase, Quadrupole Effects in Solid-State Nuclear Magnetic Resonance, in: H. Pfeifer, P. Barker (Eds.), Special Applications, Springer, Berlin, Heidelberg, 1993: pp. 1–90. https://doi.org/10.1007/978-3-642-50046-6_1.

[5] H. Friebolin, Ein- und zweidimensionale NMR-Spektroskopie: Eine Einführung, John Wiley & Sons, 2013.

[6] A. Schweitzer, T. Gutmann, M. Wächtler, H. Breitzke, A. Buchholz, W. Plass, G. Buntkowsky, 51V solid-state NMR investigations and DFT studies of model compounds for vanadium haloperoxidases, Solid State Nuclear Magnetic Resonance 34 (2008) 52–67. https://doi.org/10.1016/j.ssnmr.2008.02.003.

[7] O.B. Lapina, D.F. Khabibulin, A.A. Shubin, Modern solid-state NMR of quadrupolar nuclei, J Struct Chem 51 (2010) 28–46. https://doi.org/10.1007/s10947-010-0187-8.

[8] O.B. Lapina, D.F. Khabibulin, A.A. Shubin, V.V. Terskikh, Practical aspects of 51V and 93Nb solid-state NMR spectroscopy and applications to oxide materials, Progress in Nuclear Magnetic Resonance Spectroscopy 53 (2008) 128–191. https://doi.org/10.1016/j.pnmrs.2007.12.001.

[9] O.B. Lapina, V.M. Mastikhin, A.A. Shubin, V.N. Krasilnikov, K.I. Zamaraev, 51V Solid state NMR studies of vanadia based catalysts, Progress in Nuclear Magnetic Resonance Spectroscopy 24 (1992) 457–525. https://doi.org/10.1016/0079-6565(92)80008-4.

[10] J.W. Hennel, J. Klinowski, Magic-Angle Spinning: a Historical Perspective, in: J. Klinowski (Ed.), New Techniques in Solid-State NMR, Springer, Berlin, Heidelberg, 2005: pp. 1–14. https://doi.org/10.1007/b98646.

[11] R.E. Wasylishen, S.E. Ashbrook, S. Wimperis, NMR of quadrupolar nuclei in solid materials, Wiley, Hoboken, NJ, 2012.

[12] M. Mehring, Principles of High Resolution NMR in Solids, Springer, Berlin, Heidelberg, 1983. https://doi.org/10.1007/978-3-642-68756-3.

[13] J. Skibsted, N.C. Nielsen, H. Bildsoe, H.J. Jakobsen, 5iV MAS NMR spectroscopy: determination of quadrupole and anisotropic shielding tensors, including the relative orientation of their principal-axis systems, CHEMICAL PHYSICS LETTERS 188 (1992).

[14] U.G. Nielsen, H.J. Jakobsen, J. Skibsted, Characterization of Divalent Metal Metavanadates by 51V Magic-Angle Spinning NMR Spectroscopy of the Central and Satellite Transitions, Inorg. Chem. 39 (2000) 2135–2145. https://doi.org/10.1021/ic991243z.

[15] J. Skibsted, N.C. Nielsen, H. Bildsoe, H.J. Jakobsen, Magnitudes and relative orientation of vanadium-51 quadrupole coupling and anisotropic shielding tensors in metavanadates and potassium vanadium oxide (KV3O8) from vanadium-51 MAS NMR spectra. Sodium-23 quadrupole coupling parameters for .alpha.- and .beta.-NaVO3, J. Am. Chem. Soc. 115 (1993) 7351–7362. https://doi.org/10.1021/ja00069a038.

[16] B.A. Gee, A. Wong, Vanadium-51 MAS and Static NMR Studies of the Binary V2O5−WO3 System, J. Phys. Chem. B 107 (2003) 8382–8387. https://doi.org/10.1021/jp030379m.
[17] M. Yashima, T. Tsujiguchi, Y. Sakuda, Y. Yasui, Y. Zhou, K. Fujii, S. Torii, T. Kamiyama, S.J. Skinner, High oxide-ion conductivity through the interstitial oxygen site in Ba7Nb4MoO20-based hexagonal perovskite related oxides, Nat Commun 12 (2021) 556. https://doi.org/10.1038/s41467-020-20859-w.
[18] L.A. Al-Hajji, M.A. Hasan, M.M.I. Zaki, Kinetic and characterization studies of the formation of barium monomolybdate in equimolar powder mixture of BaCO3 and MoO3, Journal of Materials Research 18 (2003) 2339–2349. https://doi.org/10.1557/JMR.2003.0328.
